# An Integrative Approach to Study Structural and Functional Network Connectivity in Epilepsy Using Imaging and Signal Data

**DOI:** 10.3389/fnint.2020.491403

**Published:** 2021-01-12

**Authors:** Sarah J. A. Carr, Arthur Gershon, Nassim Shafiabadi, Samden D. Lhatoo, Curtis Tatsuoka, Satya S. Sahoo

**Affiliations:** ^1^Department of Neurology, School of Medicine Case Western Reserve University, Cleveland, OH, United States; ^2^Neuroimaging Department, Institute of Psychiatry, Psychology and Neuroscience, King's College London, London, United Kingdom; ^3^Department of Population and Quantitative Health Sciences, School of Medicine, Case Western Reserve University, Cleveland, OH, United States

**Keywords:** epileptic seizure networks, diffusion tensor imaging, stereotactic EEG, functional connectivity, structural connectivity, hierarchical clustering, integrative brain network analysis

## Abstract

A key area of research in epilepsy neurological disorder is the characterization of epileptic networks as they form and evolve during seizure events. In this paper, we describe the development and application of an integrative workflow to analyze functional and structural connectivity measures during seizure events using stereotactic electroencephalogram (SEEG) and diffusion weighted imaging data (DWI). We computed structural connectivity measures using electrode locations involved in recording SEEG signal data as reference points to filter fiber tracts. We used a new workflow-based tool to compute functional connectivity measures based on non-linear correlation coefficient, which allows the derivation of directed graph structures to represent coupling between signal data. We applied a hierarchical clustering based network analysis method over the functional connectivity data to characterize the organization of brain network into modules using data from 27 events across 8 seizures in a patient with refractory left insula epilepsy. The visualization of hierarchical clustering values as dendrograms shows the formation of connected clusters first within each insulae followed by merging of clusters across the two insula; however, there are clear differences between the network structures and clusters formed across the 8 seizures of the patient. The analysis of structural connectivity measures showed strong connections between contacts of certain electrodes within the same brain hemisphere with higher prevalence in the perisylvian/opercular areas. The combination of imaging and signal modalities for connectivity analysis provides information about a patient-specific dynamical functional network and examines the underlying structural connections that potentially influences the properties of the epileptic network. We also performed statistical analysis of the absolute changes in correlation values across all 8 seizures during a baseline normative time period and different seizure events, which showed decreased correlation values during seizure onset; however, the changes during ictal phases were varied.

## Introduction

Brain connectivity measures are widely used to study and characterize both normal brain functions and changes that occur in serious neurological disorders such as Alzheimer's disease and epilepsy (Bettus et al., 2010; van Diessen et al., 2012; Burggren and Brown, 2014; Bartolomei et al., 2017). Although traditional approaches for analyzing brain connectivity have often exclusively used either functional connectivity measures or structural brain connectivity measures, there are an increasing number of studies that use these two complementary measures together (Guye et al., 2010; Tertel et al., 2011). Structural connectivity measures derived from diffusion weighted imaging (DWI) data represent stable white matter tracts. Functional connectivity measures derived from functional imaging data or electrophysiological signal data represent coupling between brain activity recorded from different regions (Friston, 2011).

Although, structural and functional networks represent complementary views of brain connectivity, accurate characterization of the interactions between these two types of networks is difficult. For example, certain brain regions are highly connected by white matter tracts and have stable long-term functional connections between them. However, transient connections also exist and functional connectivity can change from moment to moment depending on the state of the brain and the task being performed (Bassett et al., 2011; Hutchison et al., 2013; Di and Biswal, 2015). In addition, structural network connections are capable of slowly changing over time as new skills are learned or brain injury/pathology appears and forces the formation of new pathways (Meier et al., 2016; Voss et al., 2017). Therefore, a better understanding of the interaction between structural and functional networks, especially in severe neurological disorders, can provide important insights into the progression and evolution of these diseases.

Functional and structural connectivity studies have revealed differences in a wide range of neurological conditions. For example, using resting-state magnetoencephalography (MEG) data, differences in functional connectivity were found in obsessive compulsive disorder patients as compared to healthy controls (Koh et al., 2018). Similarly, changes detected in white matter structural connectivity of epilepsy patients have been used to differentiate epilepsy patients with and without cognitive degeneration (Vaessen et al., 2011) and resting-state functional network analysis has been used to evaluate seizure networks in presurgical evaluation of medial temporal lobe epilepsy (Bettus et al., 2010). In this article, we describe the development of an integrative approach to characterize the dynamics of network motifs formed during epileptic seizures using functional connectivity and correlate the results with structural connectivity measures derived from pre-surgical DWI.

Epilepsy is a serious neurological disorder affecting more than 60 million persons worldwide with disruption of brain networks that may or may not involve the presence of brain pathology (Berg et al., 2010; Richardson, 2012; Chowdhury et al., 2014). Similar to other brain connectivity research studies, there is increasing interest in using network analysis techniques to characterize epileptic networks where brain locations are represented as nodes and association between these nodes (fiber tracts or functional correlation) are represented as edges (van Diessen et al., 2012). Network analysis of brain connectivity data provides important insights into the network organization of brain regions at both local and global levels (Rubinov and Sporns, 2010; Kramer and Cash, 2012). There has been significant work in the use of network analysis methods to study epileptogenic zone extent using functional network data (Ponten et al., 2007; Kramer and Cash, 2012; van Diessen et al., 2012; Bartolomei et al., 2013, 2017). In addition, many studies have characterized changes in the structural networks of epilepsy patients, including cortical and subcortical atrophy (McDonald et al., 2008; Bonilha et al., 2010; Otte et al., 2012; Whelan et al., 2018).

### Computation of Functional and Structural Connectivity Measures in Epilepsy

Functional connectivity measures to study epileptic network are often derived from electroencephalography (EEG) data recorded from scalp electrodes, subdural electrodes, and depth electrodes (Bartolomei et al., 2017). In particular, stereotactic EEG (SEEG) recorded using intracranial depth electrodes provide fine granularity signal data that are not affected by barriers, such as scalp or dura mater between the electrode and site of electrophysiological event (Rosenow and Lüders, 2001). During evaluation for surgical resection in focal epilepsy, SEEG data is recorded from intracranial depth electrodes to localize seizure foci. Additionally, high resolution magnetic resonance (MR) imaging is typically carried out before implantation to assist with surgical planning. Diffusion weighted images, in particular, aid in the planning of surgical resection to minimize damage to major white matter structural pathways. However, diffusion weighted images can be used to gain more detailed information and a number of different structural connectivity metrics have been developed. Most commonly used metrics are the fractional anisotropy (a measure of the direction of diffusion) and mean diffusivity (how much water has diffused in a particular region). The additional application of tensors (Basser et al., 2000), or other mathematical constructs (Behrens et al., 2003), allow for a voxel-wise estimation of the overall direction of diffusion.

This detailed information enables white matter pathways to be traced from voxel-to-voxel throughout the brain and the major tracts to be reconstructed. Although probabilistic tracking algorithms provide a higher probability of the tracts being true as compared to deterministic tracking, they are still subject to the same limitations as deterministic tracking. Diffusion images are typically acquired at a resolution of a few millimeters and each voxel will contain many white matter fibers, often orientated in many different directions. Therefore, tracking algorithms may struggle to resolve crossing or touching fibers within the individual voxels. The application of techniques such as spherical deconvolution are able to improve the estimation of directionality by allowing multiple directions to exist (Alexander et al., 2002). In this paper, we use the electrode contacts used for recording SEEG as end points to compute fiber tracts between the electrode contacts and analyze the resulting structural connectivity network together with functional network information.

There are several significant challenges for integrative analysis of epileptic network, including: (1) the large volume of SEEG data that need to be processed and mapped to specific events (e.g., seizure onset, ictal phases); and (2) computation of white matter fiber tracts from structural and diffusion MRI data at different resolutions and in a common anatomical space without degradation of the data. In this paper, we describe the development of an integrative analysis approach for epileptic networks using seizure events as a frame of reference and the use of a new workflow-based tool for efficient processing as well as analysis of SEEG data. We describe the graph motifs formed during 27 seizure events using clustering-based network analysis and correlate these functional connectivity features with the structural connectivity measures. In addition, we perform statistical analysis of the changes in coupling measures between brain regions during multiple events, including ictal events. Ictal events occur during seizure and they include phases (labeled as ictal 1 phase, ictal 2 phase etc.) representing propagation of abnormal activity to other parts of the brain following seizure onset.

### Related Work

We performed a PubMed search in August 2018 with keywords (stereotactic EEG and tractography) OR (SEEG and tractography) and the result included only two publications. One of the publications described the use of intraoperative MRI and functional navigation to address surgical challenges associated with tumors located in the eloquent brain regions (Sommer et al., 2014). The second publication describes the creation of a database to compare deep brain stimulation target areas using a common coordinate system (Höflich et al., 2013). However, some studies have described the correlation of electrocorticography (ECoG) and diffusion MRI to characterize structural connectivity measures in the context of ECoG data. For example, a study by Swann et al. describes the use of task-based ECoG data, diffusion MRI, macrostimulation, and cortico-cortical evoked potentials (CCEPs) to study the network control between pre-supplementary motor area (preSMA) and right inferior frontal gyrus (rIFG) (Swann et al., 2012). A study that is similar to our work presented in this paper used ECoG constrained tractography to correlate structural connectivity derived from DTI data with ECoG data recorded during a working memory task (Tertel et al., 2011).

In contrast, our study uses functional connectivity networks formed during seizure events using SEEG data, which has a higher resolution than ECoG, and explores the correlation between the functional network and structural connectivity measures. In addition, our study leverages a new informatics tool called the Neuro-Integrative Connectivity (NIC), which was developed by us to process and analyze large-scale EEG data (Sahoo et al., 2020). To the best of our knowledge, there are no existing informatics tools that use a workflow system to process SEEG signal data for automated computation of functional connectivity measures and network analysis of epileptic networks (Mouček et al., 2014; Bigdely-Shamlo et al., 2016). Further, there has been limited work in the development of an interoperable data representation format for electrophysiological signal data. In our previous work, we developed the Cloudwave Signal Format (CSF) (Jayapandian et al., 2015), which is used as a common abstraction model for signal data in the NIC platform for the computation of signal coupling measures and for network analysis.

Various measures have been proposed to characterize the coupling between signal data recorded from different brain regions during clinical events, including linear or non-linear correlation measures using signal amplitude and coherence measures based on signal frequency (Bartolomei et al., 2017). In particular, non-linear correlation coefficient values have been shown to be effective in characterizing functional connectivity between brain regions with the ability to derive directionality of connections (Pijn et al., 1989; Pijn and da Silva, 1993). These correlation values together with directionality information are increasingly being used to generate directed graph networks, which can be used to study functional networks (Kramer and Cash, 2012; Stam and van Straaten, 2012; van Diessen et al., 2012). Similarly, structural network changes in epilepsy have also been studied using network analysis measures (Besson et al., 2014; Abdelnour et al., 2015).

We derive structural connectivity from DWI tractography between contacts of depth electrodes involved in recording seizure events. There are many tools for processing DWI data and for deriving deterministic as well as probabilistic measures corresponding to structural networks, for example FMRIB (Functional Magnetic Resonance Imaging of the Brain) Software Library (FSL) Diffusion Toolbox diffusion module, Diffusion Spectrum Imaging (DSI) Studio, Camino and MRtrix. However, there has been limited work in integrative analysis of signal data from SEEG electrodes and DWI data for characterizing the properties of epileptic networks as described in this paper.

## Materials and Methods

This study analyses SEEG and DWI data from a patient with diagnosis of refractory left insula epilepsy of 5 years duration with normal developmental history. The patient suffered 4–10 seizures per day and underwent routine evaluation for surgical resection of the seizure generating tissues. This included routine pre-surgical MRIs and computer tomography (CT) before depth electrodes were stereotactically implanted in the insula, opercular and mesial temporal regions of both the right and left hemispheres (we refer to Lacuey et al., 2015, 2016 for more details). Prior to conducting the study, ethical approval was obtained from the Internal Review Board at University Hospital Cleveland Medical Center (UHCMC), Cleveland, Ohio. As a retrospective chart review study of anonymized data, informed consent was not required. Retrospective anonymized clinical data, including MRIs, CTs, depth electrode recordings and patient notes, were retrieved from the UHCMC Epilepsy Center.

**Figure 1:**
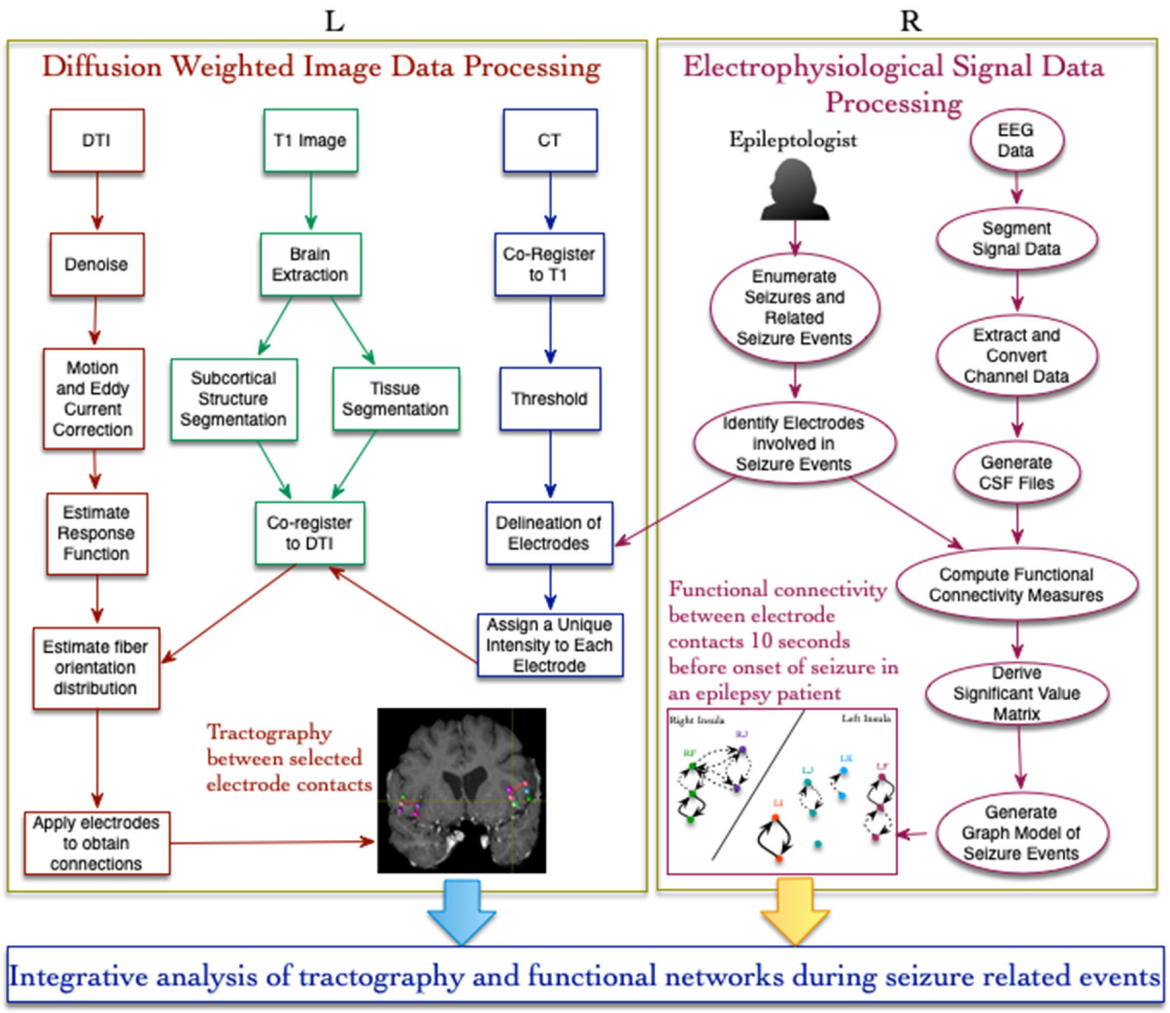
Workflow-based approach for integrative analysis of structural (L) and functional (R) connectivity networks in epilepsy. The structural connectivity workflow (on the left) involves image processing, registration, and use of electrode contacts as constraints for fiber tracting. The functional connectivity workflow (on the right) uses a new signal data format called CSF for computing coupling measures between signal recorded from different brain locations.

The lead location of the depth electrodes was confirmed via CT images acquired within 24 h of insertion. The electrodes consisted of platinum-iridium contacts with a diameter of 1.1 mm by 2.5 mm and each lead was 31 cm long with 10–12 electrodes that were placed every 5 mm, beginning from the tip. Based on their role in recording seizure activity (identified during EEG reading session), 15 electrodes were selected for analysis in this study and the details of these electrodes are provided in Table 1. A detailed description of the seizure events, the timeline, list of electrode contacts involved, and type of events across 8 seizures recorded in the patient during the stay in the epilepsy center are provided in Table 2. In the following sections, we describe the method used for data acquisition, processing, and analysis of both imaging and SEEG signal data using a workflow-based tool (Figure 1).

**Table 1:** Location of electrodes of interest.

**Electrode**	**Location**
RF1-3	Perisylvian/opercular region
RJ1-2	Posterior short gyrus of the anterior insula
LF1-3	Perisylvian/opercular region
LI1-3	Perisylvian/opercular region
LJ1-3	Middle short gyrus of the anterior insula
LK1-2	Posterior short gyrus of the anterior insula

*R, right hemisphere; L, left hemisphere. F, I, J, and K refer to the lead within that hemisphere. The number represents the electrode location along the lead, counting from the tip*.

**Table 2:** Details of seizure events recorded during stay of patient in epilepsy monitoring unit.

**Seizure ID**	**Onset frequency**	**Duration**	**Seizure onset**	**Ictal 1 phase**	**Ictal 2 phase**	**Ictal 3 phase**
1	Rhythmic delta 4–5 Hz	45 s	LJ1-3, LK1-2	LF 1-3, RF1-3		
2	Rhythmic delta 4–5 Hz	56 s	LJ 1-3, LK1-2	LF1-3, RF1-3	RJ 1-2	LI 1-2
3	Rhythmic delta 4–5 Hz	58 s	LJ 1-3, LK1-2	RF 1-3	LF 1-3, RJ 1-2	LI 1-2
4	Rhythmic delta 4–5 Hz	42 s	LJ1-3, LK 1-2	LF 1-3, RF1-3, RJ 1-2	RJ 1-2	LI 1-2
5	Rhythmic delta 4–5 Hz	49 s	LJ 1-3, LK 1-2	LF 1-3, RF 1-3	LI 1-2	
6	Rhythmic delta 4–5 Hz	50 s	LJ 1-3, LK 1-2	LF 1-3, RF 1-3	RJ 1-2	LI 1-2
7	Rhythmic delta 4–5 Hz	44 s	LJ 1-3, LK 1-2	LF 1-3, RF 1-3, RJ 1-2	LI 1-2	
8	Rhythmic delta 4–5 Hz	43s	LJ 1-3, LK 1-2	LF 1-3, RF1-3, RJ 1-2	LI 1-2	

### Imaging Data Acquisition and Processing

Brain MRI images for surgical planning were acquired with a 3T MR scanner, these included: (1) a high resolution T1-weighted MPRAGE scan with gadolinium contrast, using a 288 by 384 field of view (FOV) yielding an in-plane resolution of 0.67 × 0.67 mm^2^ and 1 mm thick slices, a total of 160 slices were acquired; and (2) 30-direction diffusion weighted image with a FOV of 128 × 128 producing a final in-plane resolution of 1.8 × 1.8 mm^2^ and 3 mm slices. Full-brain coverage was achieved with 64 slices. One B_0_ image was acquired, and the b factor was 1,000. A post-surgical brain CT image was also acquired for localization of electrode positions, the FOV was 512 × 512 producing a voxel resolution of 0.98 × 0.98 × 1 mm^3^ with 237 slices.

#### CT

The post-surgical CT image was co-registered to the T1 image using FSL's FMRIB Linear Image Registration Tool (FLIRT) module (Jenkinson and Smith, 2001; Jenkinson et al., 2002) and a rigid-body transform with 6 degrees of freedom was used. Once in T1 space, the CT image was thresholded to isolate the electrodes. They appeared as very bright artifacts, with a value much higher than the rest of the brain structures. Once isolated, FSL's image viewer was used to manually delineate each individual electrode of interest and create a separate NIfTI (Neuroimaging Informatics Technology Initiative) image for each one. Each electrode image was multiplied by a unique number from 1 to 15 and then all electrodes were added together to form a single image. The numbers were assigned randomly as the purpose was to create a unique image intensity value for each electrode so they could be distinguished in the final single image using the software. The final image was used in the last stage of the DTI analysis below. The transformation matrix from T1 space to DTI space was determined using FSL's flirt command and applied to the image header only. Only the voxel grid is translated, not the image itself. This allowed the regions of interest (ROIs), defined in T1 space, to be translated into DTI space without altering the image intensities or electrode sizes.

#### T1

The T1 weighted image containing the T1 to DTI space transform was initially brain extracted using FSL (Smith, 2002). Segmentation of gray, white matter, cerebrospinal fluid and subcortical structures was performed using FSL's FMRIB Automatic Segmentation Tool (FAST) (Zhang et al., 2001) and FIRST (Patenaude et al., 2011) functions implemented through the MRtrix3 software (www.mrtrix.org). FAST is a fully automated segmentation module that uses Markov random field modeling. FMRIB Integrated Registration and Segmentation Tool (FIRST) segments the subcortical structures using a Bayesian framework to compare the size and shape of intensity regions to a training set of structures. A composite image was then composed to inform and anatomically constrain the DTI tractography (Smith et al., 2012).

#### DTI

The diffusion weighted images were pre-processed using both FSL and MRtrix. Initially, MRtrix's denoising function was applied (Veraart et al., 2016a,b). This creates a noise map based on the PCA (principle component analysis) domain and allows for extraneous signals to be removed. Motion correction and eddy current correction was performed using FSL's “eddy” command (Andersson and Sotiropoulos, 2016) implemented through the “dwipreproc” script in MRtrix. A whole brain mask was applied to remove voxels outside the brain before estimating the response functions for spherical deconvolution using the Tournier algorithm. This is an iterative algorithm for single fiber voxel selection (Tournier et al., 2013). Finally, the fiber orientation distribution was estimated using constrained spherical deconvolution (Tournier et al., 2008). This allows the direction of the water motion to be estimated as a composite of directions and it is able to detect crossing fibers more readily than the tensor calculations typically used in DTI analyzes.

Whole brain probabilistic tractography was performed in MRtrix using second order integration over the fiber orientations distributions (iFOD2 algorithm) (Tournier et al., 1992). The tracking was anatomically constrained using the segmented composite T1 image described in the previous section. This prevents tracking into gray matter and areas of cerebrospinal fluid. The electrode image was then applied to the resulting tracks to determine the number of connections that existed between each electrode and every other electrode.

### Signal Data Processing and Analysis

#### Signal Data Processing

SEEG data was acquired from the 15 depth electrodes described earlier and data was stored in files using the European Data Format (EDF) (Kemp et al., 1992; Kemp and Olivan, 2003; Kemp and Roessen, 2013). To address the challenges associated with using EDF files to derive functional connectivity measures, CSF files were generated from the EDF files using the NIC tool. The NIC tool features a function to generate CSF files from EDF files with user-defined parameters, for example duration of signal data segment stored in a CSF file, and number of segments stored in a CSF file. In this study, the EEG data recorded from the patient was stored in 12 EDF files and these EDF files were processed using the NIC tool to generate 204 CSF files. These CSF files were subsequently processed by the NIC tool to derive non-linear correlation coefficient values as a measure of functional connectivity (Pijn et al., 1989).

#### Derivation of Graph Model of Functional Networks

There are multiple measures of functional connectivity that are based on the degree of synchronization between brain activity recorded over time either during task-based or resting-state events (van Diessen et al., 2012). In epilepsy, functional connectivity measures derived from signal data are based on an assumption of linear correlation (Brazier, 1983; Pijn and da Silva, 1993) or coherence of signal frequency (Brazier, 1983; Da Silva and Mars, 1987), or non-linear correlation (Marshall et al., 1983; Pijn et al., 1989). In this paper, we use a non-linear correlation coefficient measure h^2^ developed by Pijn et al. that is based on estimation of amplitude y of signal Y from amplitude x of signal X assuming predicted value of y for given value of x is a regression curve (Pijn et al., 1989). The non-linear correlation coefficient h^2^ is asymmetric, that is ${\text{h}}_{\left(\text{x}|\text{y}\right)}^{2}$ ≠ ${\text{h}}_{\left(\text{y}|\text{x}\right)}^{2}$, which allows us to derive directionality together with strength of coupling between two signal values.

To generate a more concise representation of the correlation values, significant values were computed using a threshold value derived from the average and standard deviation of h^2^ values computed using signal data recorded 20 s before the onset of seizure (selected by a domain expert). This threshold value was computed once and consistently used for computing the significant values in all 27 events across 8 seizures. The temporal bin for individual h^2^ values corresponds to the duration of clinically defined and distinct events, including seizure onset, ictal 1 phase, ictal 2 phase etc. The significant values together with directionality information from the h^2^ values were used to generate directed graph models for all the 8 seizures recorded from the patient during the patient's stay in the epilepsy monitoring unit. The graph model consisted of nodes corresponding to electrode contacts and edges corresponding to interactions between the nodes. The significant values derived from the non-linear correlation values are modeled as directed edges with solid edges representing values >2 standard deviations and dashed edges representing values >1 standard deviation from baseline, respectively (using an approach similar to Wendling et al. for computing significant values Wendling et al., 2009). Using this directed graph network model, we computed hierarchical clustering measure to characterize the organization of brain regions involved in epileptogenicity during seizure onset and ictal phases (corresponding to propagation of abnormal brain activity to other parts of the brain following seizure onset).

#### Characterizing the Formation of Graph Motifs in Seizure Network Using Hierarchical Clustering

Several studies on brain connectivity have shown a hierarchical organization of brain networks for both human and non-human subjects (Bassett et al., 2010; Stam, 2014). In epilepsy, various studies have identified the formation and destruction of network structures during epileptic seizure (Kramer and Cash, 2012). Kramer et al. used ECoG signal data to derive functional connectivity networks and identified a dominant subnetwork that formed during seizure, which disbanded toward the end of the seizure (Kramer et al., 2010). In this paper, we use the hierarchical clustering method to detect “communities of brain regions” (corresponding to electrode contacts) that form during seizure events by clustering nodes based on their similarity measures (Newman, 2018). We used cosine similarity measure in this paper to compute the similarity between all vertices in the directed graphs corresponding to 27 events, including seizure onset and ictal phases, across 8 seizures.

The cosine similarity is measured as the dot product between the rows of the adjacency matrix corresponding to a pair of vertices (Salton, 1989). Given two vertices x, y, and M_xk_ and M_ky_ are the rows of the adjacency matrix M representing the directed graph of a seizure event, the cosine similarity is computed as:

$$\begin{array}{l} \sigma_{\text{xy}}=\frac{\sum_{k}M_{xk}M_{ky}}{\sqrt{\sum_{k}M_{xk}^{2}}\sqrt{\sum_{k}M_{ky}^{2}}} \end{array}$$

(Newman, 2018). Once the cosine similarity values are measured for all pair-wise vertices, we generate the hierarchical clustering by combining vertices with the highest similarity followed by aggregating groups of vertices, again, using their similarity values. These steps are repeatedly applied until all the vertices are aggregated into a single group. The resulting hierarchical clusters are represented as dendrograms, which allows easy interpretation of the formation of groups in the vertices corresponding to electrode contacts.

#### Evaluation of Correlation Changes During Seizure Events

The modules formed during seizure events (described in the previous section) represent patterns of coupling between brain regions that occur during seizure events. Previous studies have found that seizures events are not associated with consistently hypersynchronous state (Kramer et al., 2010) and that there is increased degree of coupling during seizure onset followed by decreased coupling during ictal phases (Bartolomei et al., 2001, 2017). However, there is limited data available to systematically characterize the changes in correlation values that occur during seizure events as compared to the inter-ictal period. To determine whether these correlation patterns vary significantly from the inter-ictal period, we performed a signed-rank test using a pre-ictal time duration as baseline normative event, which was identified during EEG reading by a research fellow. We evaluated the changes in correlation values between contacts on distinct electrodes during seizure onset and subsequent ictal phases as compared to the baseline event.

Using the correlation data for each pair of contacts that are not on the same electrode, we generated a vector of h^2^ values for the seizure onset and subsequent ictal phases in each seizure. The difference values of the correlation values at each seizure stage were computed by subtracting corresponding h^2^ values computed during a control period non-seizure activity as described above. This control period served as a baseline reference for h^2^ values for each pair of electrode contact. The sign-rank test was used to assess whether the respective differences in the stage vs. control period h^2^values per electrode pair are significantly different than zero, with the seizures providing replication of observation. Two-sided *p*-values were computed using Matlab software (The MathWorks, Inc.) In addition, to adjust for multiple comparisons, we employed the Benjamini-Hochberg (B-H) false discovery rate method with a false discovery rate of 0.05 to compute the B-H critical value (Benjamini and Hochberg, 1995). The original *p*-values and corresponding adjusted B-H *p*-value cutoffs are listed in the (Supplementary Tables 1–4). Results are interpreted relative to the adjusted thresholds. We discuss the results in the next section.

## Results

### Functional Connectivity and Modularity Analysis

**Figure 2:**
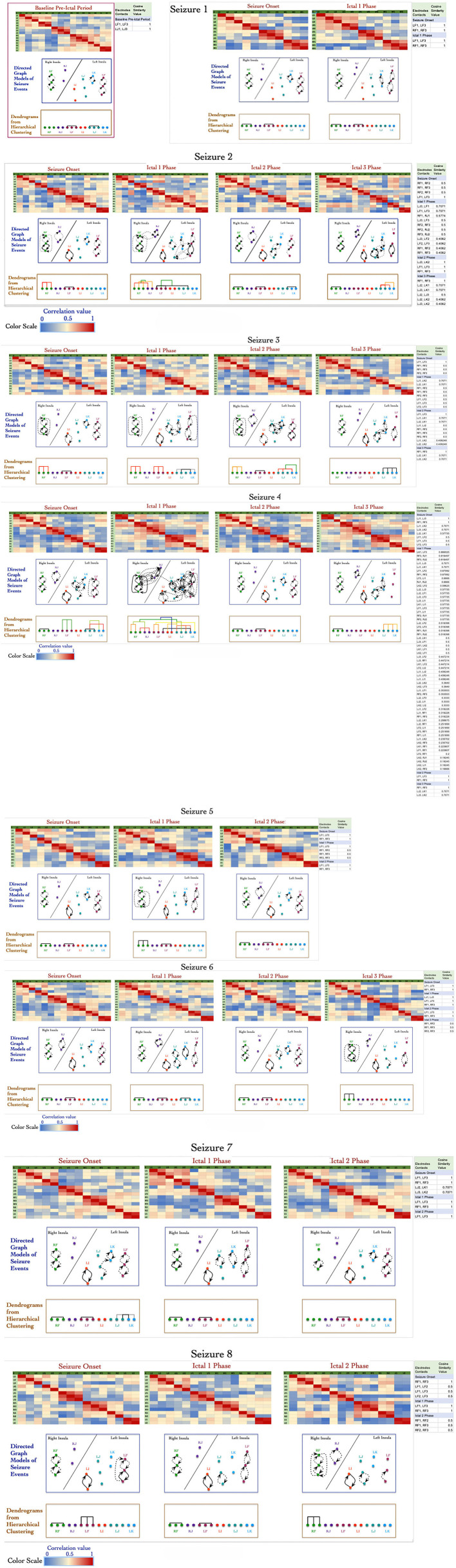
Adjacency matrix representation of correlation values between electrode contacts during 27 events across 8 seizures derived from non-linear correlation coefficient measures using color to represent strength of coupling (red denotes stronger correlation and blue denotes weaker correlation). The directed graph structures derived from the correlation matrices using significant values with dashed lines representing lower and solid lines representing higher values. The modularity of the vertices in the graph are characterized using hierarchical clustering and visualized as dendrograms.

Figure 2 shows a panel view of: (1) correlation values (represented as heat map) that were computed for 27 events; (2) the directed graph structures derived from the correlation values; and (3) dendrograms showing formation of graph motifs during seizure onset and subsequent ictal phases. The directed graph models for the 27 events show that a variety of graph motifs are formed during seizure onset with additional motifs being formed as well as disintegrating during subsequent ictal phases.

Similar to results reported in earlier work by Kramer et al. using undirected graph models (Kramer et al., 2010), the directed graph motifs in Figure 2 are formed during the seizure onset phase with a significant number of connections occurring during ictal 1 phase. There are common patterns in terms of the subgraph motifs formed during events across the 8 seizures, for example the contacts on electrodes LF and RF form highly connected subgraphs during seizure onset across the 8 seizures (Figure 2). During seizure 2, seizure 3, and seizure 4, there are multiple instances of formation of cliques or all-to-all connected graph motifs involving electrodes in perisylvian/opercular, short gyrus of the anterior insula. These densely interconnected graph motifs disintegrate during ictal 2 phase in most of the seizures with the graph network showing similarities to the sparse interconnections of the baseline normative event in Figure 2.

During ictal 3 phase in seizure 2, 3, and 4, there is re-formation of highly interconnected graph motifs. However, these graph motifs formed during ictal 3 phase do not show the high level of interconnectivity that are seen during seizure onset or ictal 1 phase. In this patient, there are no consistent patterns of graph motifs in events, such as seizure onset, ictal 1 phase, and ictal 2 phase, across all the 8 seizures, which makes it challenging to apply network analysis measures to infer seizure onset zone for this patient.

Figure 2 also shows the formation of a connected motifs among the vertices represented as dendrograms, which were computed based on the hierarchical clustering method using cosine similarity measures of group vertices. The x-axis of the dendrograms (leaves) consists of the electrode contacts and the level at which two sets of vertices merge along the y-axis. The y-axis represents the dissimilarity between two sets of vertices. The color of the connecting edges in the dendrograms represents the similarity between the sets of vertices, which is computed using cosine similarity measure as described in the previous section Signal Data Processing and Analysis. Figure 2 shows that there is maximum activity related to merging of different sets of vertices during seizure onset and ictal 1 phase followed by ictal 3 phase with limited or no formation of clusters during ictal 2 phase. For example, during seizure onset phase in seizure 4 the electrode contacts on LJ (in middle short gyrus) and LK (in posterior short gyrus) progressively form connected clusters.

During ictal 1 phase of the same seizure (seizure 4), the electrode contacts in each insula form clusters of graph motifs before merging with clusters of other graph motifs across the two insula. It is interesting to note that electrode contacts LF3 (perisylvian/operculum region of the left insula) and LK1 in the posterior short gyrus of the left insula have higher similarity compared to adjacent contacts on the same lead, that is, LF1, LF2, and LK2. Similarly, in the right insula the electrode contact RF3 has higher similarity with electrode contacts RJ1 and RJ2 as compared to contacts on the same electrode, that is, RF1 and RF2. The formation of clusters declines significantly during ictal 2 and ictal 3 phases in seizure 4.

**Figure 3:**
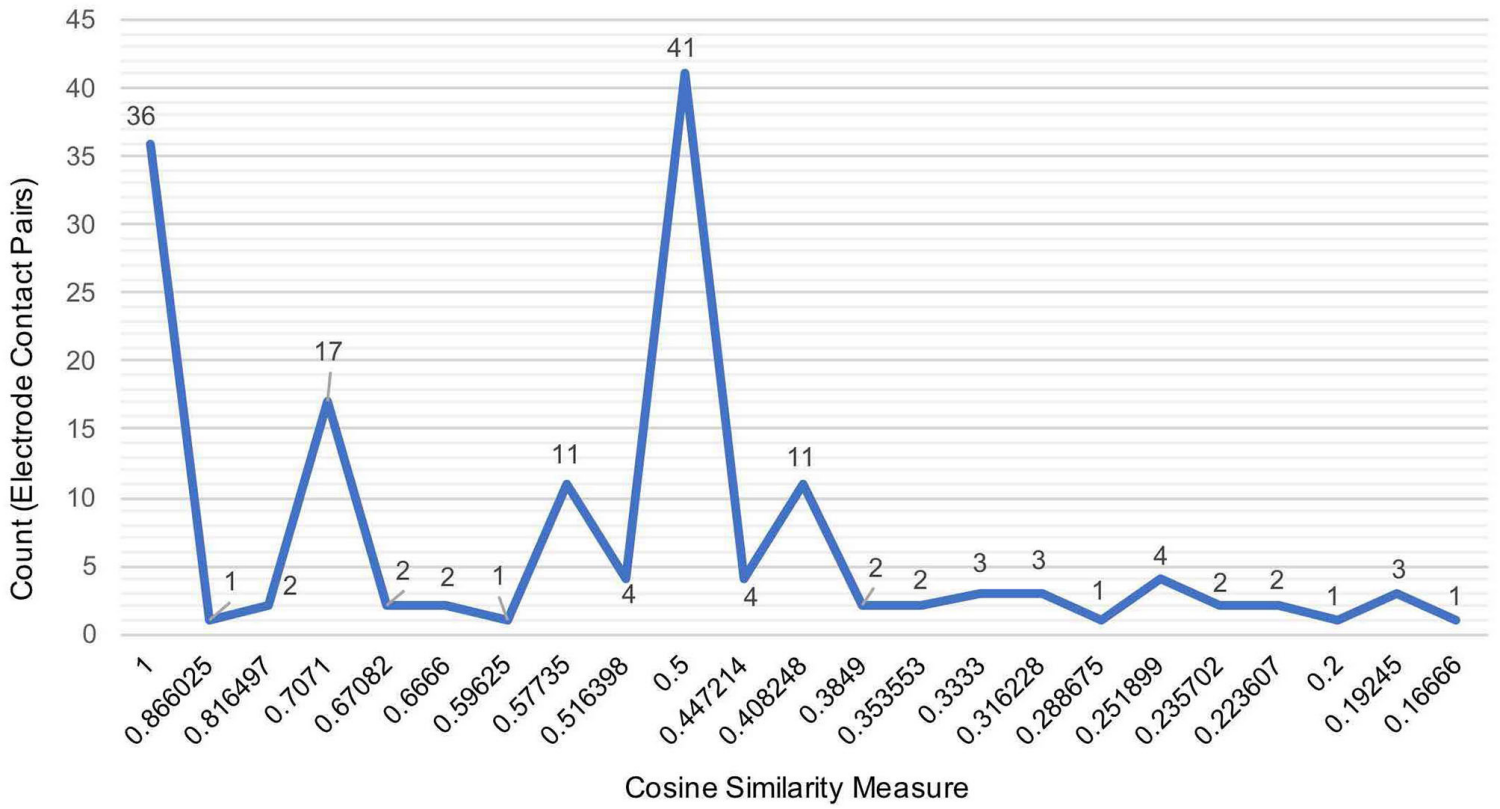
The distribution of the aggregated count of electrode pairs corresponding to cosine similarity values that are used to compute clustering of brain regions in 27 events across 8 seizures to generate dendrograms shown in the previous figure.

**Figure 4:**
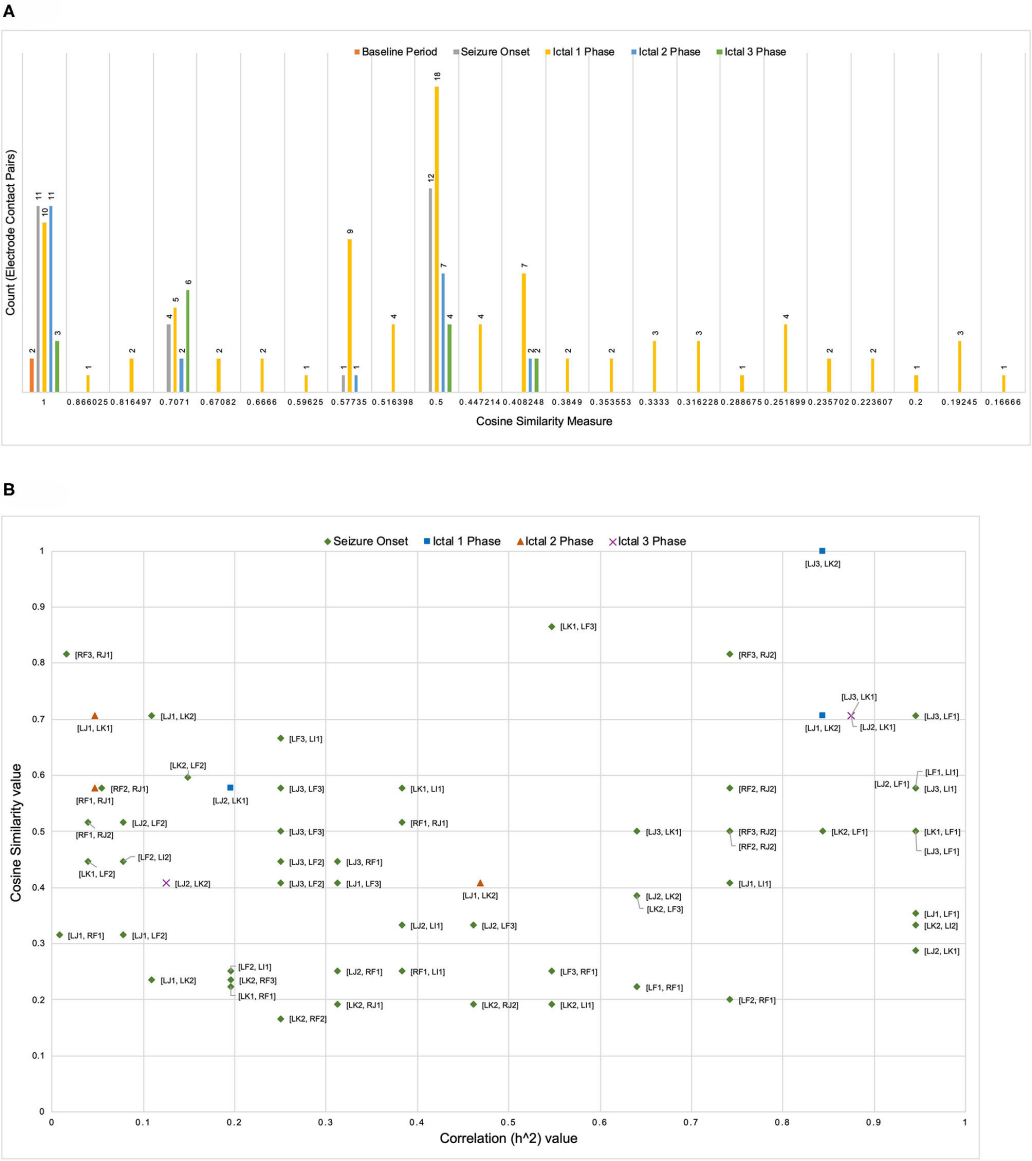
**(A)** The clustering of cosine similarity values for electrode pairs according to baseline, seizure onset, and ictal events in 27 events across 8 seizures shows significant increase in interactions between electrode pairs during ictal 1 phase as compared to any other event (including baseline event) The figure does not include a bin for cosine similarity value of 0 (a figure with bin for cosine similarity value of 0 is available as Supplementary File). **(B)** For seven pairs of electrode contacts that had non-zero cosine similarity values across multiple seizure events (in addition to ictal 1 phase) there is no association between correlation values and cosine similarity values.

Figure 3 illustrates the distribution of count of the electrode contact pairs for different values of cosine similarity measures that is used to generate the dendrograms described above. It is interesting to note that the highest and second highest count of electrode contact pairs (41 and 36, respectively) correspond to the cosine similarity measure of 1 and 0.5. These counts may be interpreted as the occurrence of a large number of interactions between brain regions with high degree of structural similarity. To gain a better understanding of the distribution of these cosine similarity measure in the baseline period as well as other seizure events, we grouped the cosine similarity values by events across all the 8 seizures. Figure 4A shows the aggregated cosine similarity values during baseline event, seizure onset, ictal 1, ictal 2, and ictal 3 phases across the 8 seizures.

Figure 4A clearly shows that there are significant differences between the baseline period and other events with ictal 1 phase in particular showing a cluster of cosine similarity values between 1 (high degree of similarity) and 0.16 (low degree of similarity). In contrast to the baseline phase, the seizure onset and ictal phases show hierarchical organization of interacting brain regions, which is consistent with previously reported results describing a hierarchical organization of connectivity between brain regions with respect to epileptogenicity (Bartolomei et al., 2017; Shine et al., 2017). The large number of electrode contacts with high cosine similarity measure of 1 during seizure onset, ictal 1, and ictal 3 phases are consistent with the proposed theory that seizures are characterized by a hypersynchronous state and that the level of synchronization decreases toward end of seizure (Kramer et al., 2008; Kramer and Cash, 2012). Table 3 lists the specific electrode contact pairs corresponding to the cosine similarity value and the seizure events during which the electrode contact pairs have a specific cosine similarity value across the 8 seizures.

**Table 3:** Grouping of electrode contact pairs by cosine similarity values with corresponding seizure events.

**Similarity**	**Electrode Pairs**	**Events**
1	[LF1, LF3]	Seizure 1 Onset; Seizure 1 Ictal 1 phase; Seizure 2 Ictal 2 phase; Seizure 3 Onset; Seizure 3 Ictal 2 phase; Seizure 4 Ictal 2 phase; Seizure 5 Onset; Seizure 5 Ictal 1 phase; Seizure 5 Ictal 2 phase; Seizure 6 Onset; Seizure 6 Ictal 1 phase; Seizure 6 Ictal 2 phase; Seizure 7 Onset; Seizure 7 Ictal 1 phase; Seizure 7 Ictal 2 phase; Seizure 8 Ictal 1 phase
	[RF1, RF3]	Seizure 1 Onset; Seizure 1 Ictal 1 phase; Seizure 2 Ictal 2 phase; Seizure 2 Ictal 3 phase; Seizure 3 Ictal 3 phase; Seizure 4 Onset; Seizure 4 Ictal 2 phase; Seizure 4 Ictal 3 phase; Seizure 5 Onset; Seizure 5 Ictal 2 phase; Seizure 6 Onset; Seizure 6 Ictal 1 phase; Seizure 6 Ictal 2 phase; Seizure 7 Onset; Seizure 7 Ictal 1 phase; Seizure 8 Onset; Seizure 8 Ictal 1 phase
	[LJ3, LK2]	Seizure 2 Ictal 2 phase
	[LJ1, LJ3]	Seizure 4 Onset; Seizure 6 Ictal 1 phase
0.866025	[LK1, LF3]	Seizure 4 Ictal 1 phase
0.816497	[RF3, RJ1]	Seizure 4 Ictal 1 phase
	[RF3, RJ2]	Seizure 4 Ictal 1 phase
0.7071	[LJ3, LF1]	Seizure 2 Ictal 1 phase
	[LF1, LF3]	Seizure 2 Ictal 1 phase
	[LJ2, LK1]	Seizure 2 Ictal 3 phase; Seizure 3 Ictal 1 phase; Seizure 3 Ictal 2 phase; Seizure 3 Ictal 3 phase; Seizure 4 Ictal 3 phase; Seizure 7 Onset
	[LJ3, LK1]	Seizure 2 Ictal 3 phase
	[LJ1, LK2]	Seizure 3 Ictal 1 phase; Seizure 4 Onset
	[LJ1, LK1]	Seizure 3 Ictal 2 phase
	[LJ3, LK2]	Seizure 3 Ictal 3 phase; Seizure 4 Onset; Seizure 4 Ictal 3 phase; Seizure 7 Onset
	[LJ1, LJ3]	Seizure 4 Ictal 1 phase
0.67082	[LF1, LF2]	Seizure 4 Ictal 1 phase
	[RF1, RF2]	Seizure 4 Ictal 1 phase
0.6666	[LF3, LI1]	Seizure 4 Ictal 1 phase
	[RJ1, RJ2]	Seizure 4 Ictal 1 phase
0.59625	[LK2, LF2]	Seizure 4 Ictal 1 phase
0.57735	[RF1, RJ1]	Seizure 2 Ictal 2 phase
	[LJ2, LK1]	Seizure 4 Onset
	[LJ2, LJ3]	Seizure 4 Ictal 1 phase
	[LJ2, LF1]	Seizure 4 Ictal 1 phase
	[LJ3, LF3]	Seizure 4 Ictal 1 phase
	[LJ3, LI1]	Seizure 4 Ictal 1 phase
	[LK1, LI1]	Seizure 4 Ictal 1 phase
	[LF1, LF3]	Seizure 4 Ictal 1 phase
	[LF1, LI1]	Seizure 4 Ictal 1 phase
	[RF2, RJ1]	Seizure 4 Ictal 1 phase
	[RF2, RJ2]	Seizure 4 Ictal 1 phase
0.516398	[LJ2, LF2]	Seizure 4 Ictal 1 phase
	[LF2, LF3]	Seizure 4 Ictal 1 phase
	[RF1, RJ1]	Seizure 4 Ictal 1 phase
	[RF1, RJ2]	Seizure 4 Ictal 1 phase
0.5	[RF1, RF2]	Seizure 2 Onset; Seizure 3 Onset; Seizure 3 Ictal 1 phase; Seizure 3 Ictal 2 phase; Seizure 5 Ictal 1 phase; Seizure 6 Ictal 3 phase; Seizure 8 Ictal 2 phase
	[RF1, RF3]	Seizure 2 Onset; Seizure 3 Onset; Seizure 3 Ictal 1 phase; Seizure 3 Ictal 2 phase; Seizure 5 Ictal 1 phase; Seizure 6 Ictal 3 phase; Seizure 8 Ictal 2 phase
	[RF2, RF3]	Seizure 2 Onset; Seizure 2 Ictal 1 phase; Seizure 3 Onset; Seizure 3 Ictal 1 phase; Seizure 3 Ictal 2 phase; Seizure 5 Ictal 1 phase; Seizure 6 Ictal 3 phase; Seizure 8 Ictal 2 phase
	[LJ3, LF3]	Seizure 2 Ictal 1 phase
	[RF2, RJ2]	Seizure 2 Ictal 1 phase
	[RF3, RJ2]	Seizure 2 Ictal 1 phase
	[LJ2, LJ3]	Seizure 2 Ictal 3 phase
	[LF1, LF2]	Seizure 3 Ictal 1 phase; Seizure 4 Onset; Seizure 8 Onset
	[LF1, LF3]	Seizure 3 Ictal 1 phase; Seizure 4 Onset; Seizure 8 Onset
	[LF2, LF3]	Seizure 3 Ictal 1 phase; Seizure 4 Onset; Seizure 8 Onset
	[LJ1, LJ2]	Seizure 3 Ictal 2 phase
	[LJ3, LK1]	Seizure 4 Ictal 1 phase
	[LJ3, LF1]	Seizure 4 Ictal 1 phase
	[LK1, LK2]	Seizure 4 Ictal 1 phase
	[LK1, LF1]	Seizure 4 Ictal 1 phase
	[LK2, LF1]	Seizure 4 Ictal 1 phase
0.447214	[LJ3, LF2]	Seizure 4 Ictal 1 phase
	[LJ3, RF1]	Seizure 4 Ictal 1 phase
	[LK1, LF2]	Seizure 4 Ictal 1 phase
	[LF2, LI2]	Seizure 4 Ictal 1 phase
0.408248	[LJ3, LF2]	Seizure 2 Ictal 1 phase
	[LF2, LF3]	Seizure 2 Ictal 1 phase
	[RF1, RF2]	Seizure 2 Ictal 1 phase
	[RF1, RF3]	Seizure 2 Ictal 1 phase
	[LJ2, LK2]	Seizure 2 Ictal 3 phase; Seizure 3 Ictal 2 phase
	[LJ3, LK2]	Seizure 2 Ictal 3 phase
	[LJ1, LK2]	Seizure 3 Ictal 2 phase
	[LJ1, LJ2]	Seizure 4 Ictal 1 phase
	[LJ1, LF3]	Seizure 4 Ictal 1 phase
	[LJ1, LI1]	Seizure 4 Ictal 1 phase
0.3849	[LJ2, LK2]	Seizure 4 Ictal 1 phase
	[LK2, LF3]	Seizure 4 Ictal 1 phase
0.353553	[LJ1, LF1]	Seizure 4 Ictal 1 phase
	[RF2, RF3]	Seizure 4 Ictal 1 phase
0.3333	[LJ2, LF3]	Seizure 4 Ictal 1 phase
	[LJ2, LI1]	Seizure 4 Ictal 1 phase
	[LK2, LI2]	Seizure 4 Ictal 1 phase
0.316228	[LJ1, LF2]	Seizure 4 Ictal 1 phase
	[LJ1, RF1]	Seizure 4 Ictal 1 phase
	[RF1, RF3]	Seizure 4 Ictal 1 phase
0.288675	[LJ2, LK1]	Seizure 4 Ictal 1 phase
0.251899	[LJ2, RF1]	Seizure 4 Ictal 1 phase
	[LF2, LI1]	Seizure 4 Ictal 1 phase
	[LF3, RF1]	Seizure 4 Ictal 1 phase
	[RF1, LI1]	Seizure 4 Ictal 1 phase
0.235702	[LJ1, LK2]	Seizure 4 Ictal 1 phase
	[LK2, RF3]	Seizure 4 Ictal 1 phase
0.223607	[LK1, RF1]	Seizure 4 Ictal 1 phase
	[LF1, RF1]	Seizure 4 Ictal 1 phase
0.2	[LF2, RF1]	Seizure 4 Ictal 1 phase
0.19245	[LK2, RJ1]	Seizure 4 Ictal 1 phase
	[LK2, RJ2]	Seizure 4 Ictal 1 phase
	[LK2, LI1]	Seizure 4 Ictal 1 phase
0.16666	[LK2, RF2]	Seizure 4 Ictal 1 phase

In addition to analyzing the distribution of cosine similarity values, we analyzed their association with correlation values for seven pairs of electrode contacts (contacts were not on the same electrode), which has non-zero cosine similarity values across seizure events (in addition to ictal 1 phase). Figure 4B shows that high correlation values do not correspond to high cosine similarity value and vice-versa. In addition, Figure 4B shows an absence of an overall trend in the scatter plot of cosine similarity and correlation (h^2^) values. However, it is interesting to note that during both ictal 1 and ictal 3 phases, there are a number of electrode pairs with high correlation and cosine similarity value. In particular, electrode contacts on LJ (in middle short gyrus) and LK (in posterior short gyrus), that is, [LJ3, LK2], [LJ1, LK2], [LJ2, LK1], and [LJ3, LK1] have high correlation as well as cosine similarity values. In addition, it is notable that during the ictal 1 phase, only two electrode contact pairs [RF3 and RJ2] and [LJ3, LF1] have high correlation and cosine similarity values. Therefore, Figure 4B shows that electrode contact pairs with high correlation values do not form clusters of connected brain regions (detected by the cosine similarity values) during different ictal phases.

#### Sign-Rank-Test Results

**Figure 5:**
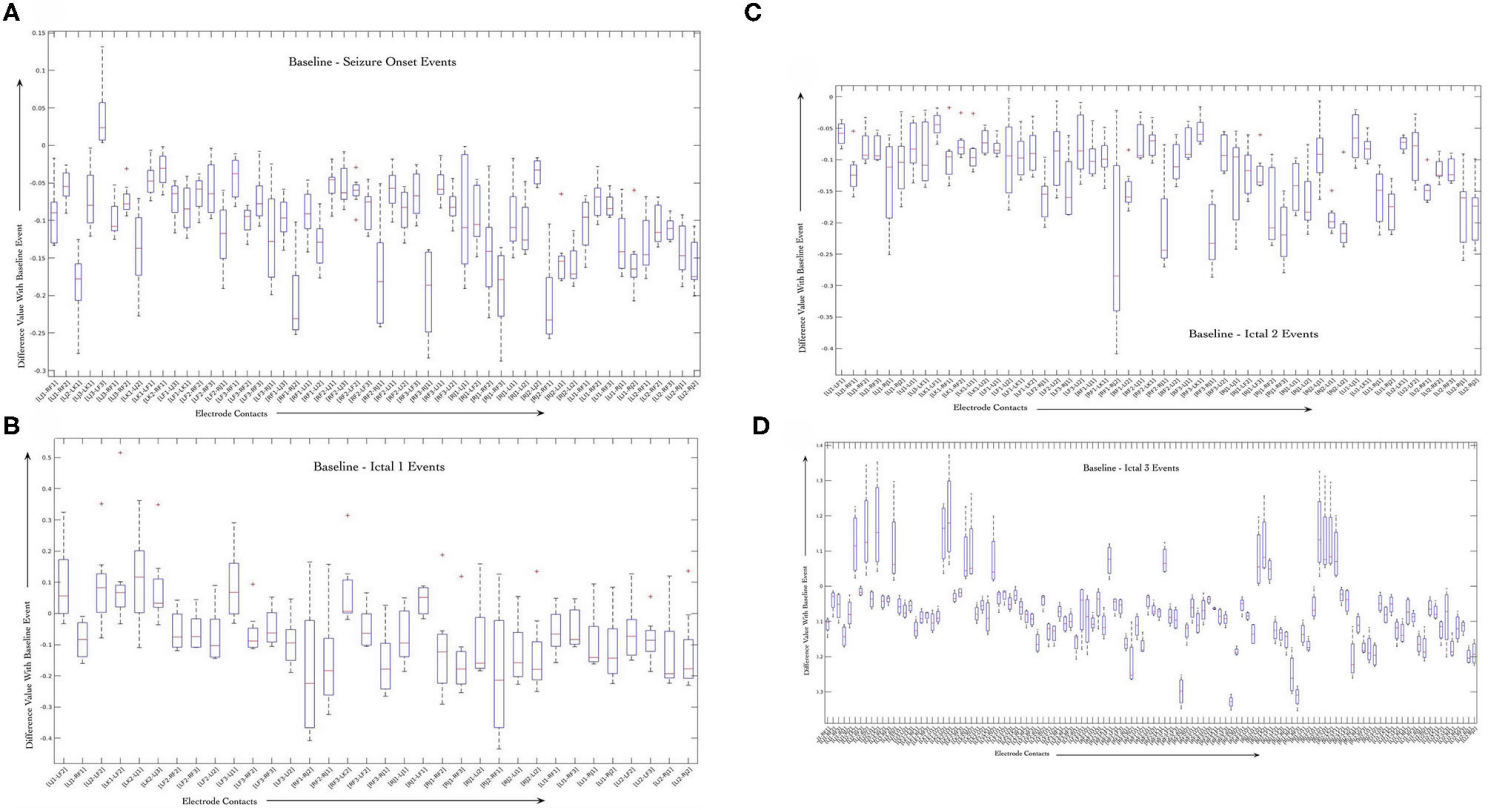
Average variability in correlation values between the baseline stage and seizure onset **(A)**, ictal 1 **(B)**, ictal 2 **(C)**, and ictal 3 **(D)** stages across all the 8 seizures. Only correlation values computed for contacts that are on distinct electrodes were used to highlight the dynamics of interactions between different brain regions during seizure events.

We performed sign-rank-test to evaluate the significance of changes in correlation values during baseline and seizure events. Figures 5A–D shows boxplots of difference between correlation values per electrode pair and for each seizure event, that is baseline as compared to seizure onset (Figure 5A), ictal 1 phase (Figure 5B), ictal 2 phase (Figure 5), and ictal 3 phase (Figure 5D) that were generated using Matlab software (MathWorks, Inc.) For each ictal stage, we present boxplots of the difference in correlation values from baseline. For a given stage, boxplots correspond to the 25 pairs with smallest *p*-values in the testing of whether or not the difference in correlation values is zero. Boxplots are based on data from all 8 seizures.

The figures show that some of the electrode contact pairs have higher degree of correlation as compared to baseline period during seizure onset (Figure 5A). However, the changes in correlation values during ictal 1 and ictal 2 phases are all negative, that is, there is decreased correlation values between electrode contacts during these two stages as compared to the baseline stage (Figures 5B,C). Finally, there is greater correlation between electrode contact pairs during ictal 3 phase represented by positive changes (Figure 5D). These results of the sign-rank-test show that there are statistically significant increases in correlation values between specific electrode contacts during seizure onset. Further, there is a trend toward decreased correlation values during ictal 1 and ictal 2 phases followed by increased correlation values during ictal 3 phase; however, these are not statistically significant. The results of the B-H false discovery rate show that the highest *p*-value that is less than B-H critical value is for the [LI1, LF2] pair of electrode contacts; therefore, the first set of 51 tests are significant during the seizure onset event (Supplementary Tables 1–4).

### Structural Connectivity

The aim of the DTI tracking was to explore the underlying structural connections that may provide more insights regarding the dynamics of the functional connectivity network observed during seizure events. No DTI tracks were found between any of the electrodes in the left insula with any electrodes in the right insula. Anatomically, white matter connections exist between the hemispheres but a direct pathway via the corpus callosum likely contains sharp bends that the MRtrix program is unable to navigate and the tracks were terminated before reaching the contralateral insula. Indirect connections may also exist via other cortical or subcortical structures. The insula is surrounded by the superior longitudinal fasciculus, the extreme capsule and the uncinate fasciculus which connect the surrounding operculum and frontal, parietal, and temporal lobes (Türe et al., 1999). White matter connections with subcortical structures have also been found, including the thalamus and amygdala (Augustine, 1996). Although waypoints were not included in this patient analysis, it is feasible to include them in further patient analysis applications of these techniques. Potential waypoints of interest may be indicated from the location of electrodes that record seizure electrical activity or from clinical opinion of seizure symptoms. The SEEG data from the patient presented here did not suggest other structures of interest to include as waypoints.

**Figure 6:**
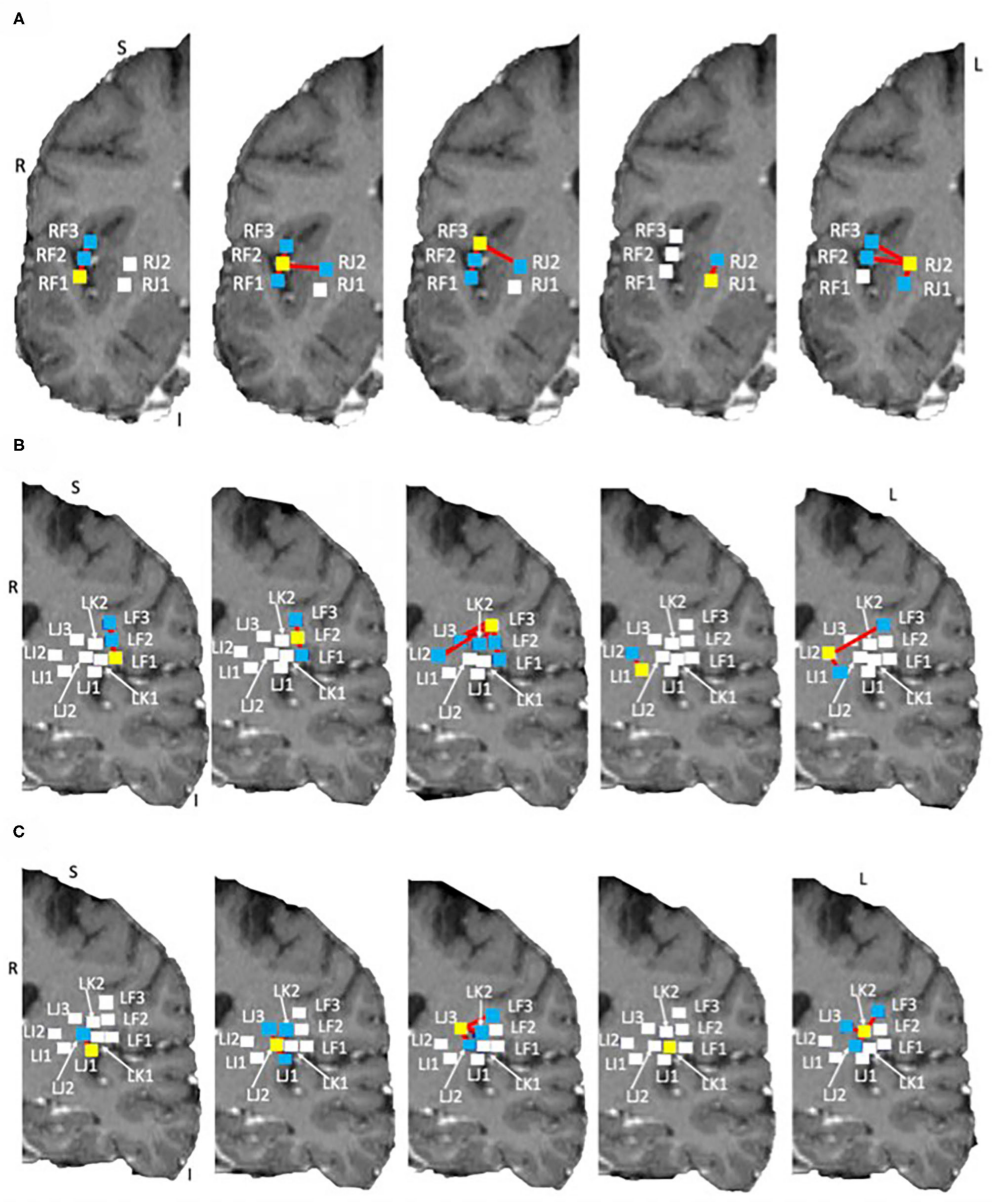
Structural networks in the right and left hemispheres overlaid on the subject own T1 in DTI space. Shown on coronal slices. The electrode of interest is denoted by a yellow square in each image. Connections to other electrodes are shown by red lines and blue squares. Electrodes with no connections are shown by white squares. **(A)** Right side networks, **(B,C)** Left side networks. Note: no connections exist for LK1, electrode shown for completeness.

Tractography connections between ipsilateral electrodes are summarized in Tables 4A,B and shown in Figures 6A,B. Within electrode connections were set to zero. Due to the absence of directionality in structural data all tracts are considered to be bidirectional. There was a strong connection (> 100 tracts) on the left side between LF1 ↔ LF2 and LI1 ↔ LI2, and RF1 ↔ RF3 and RF2 ↔ RF3 on the right side. Weaker connections were found between RF1 ↔ RF2 (75 tracts), LJ1 ↔ LJ2 (31 tracts), LJ3 ↔ LK2 (27 tracts), and LF2 ↔ LF3 (22 tracts). This pattern of structural connectivity is more closely related to the functional connectivity pattern measured before seizure onset than the activity seen during the ictal period. This suggests that the structural connections are the more established pathways that the brain uses the majority of the time and that the seizure-generated functional networks form using much weaker (not detected here) structural connections. These connections may be detectable in DWI data from a non-epilepsy person or they may be unique to the condition itself. Further studies are needed to establish healthy and diseased networks.

**Table 4:** The number of DTI tracts between the ipsilateral electrodes are shown.

**(A)**										
**Electrodes**	**RF1**	**RF2**	**RF3**	**RJ1**	**RJ2**					
RF1	0	75	108	0	0					
RF2		0	387	0	2					
RF3			0	0	2					
RJ1				0	1					
RJ2					0					
**(B)**										
**Electrodes**	**LF1**	**LF2**	**LF3**	**LI1**	**LI2**	**LJ1**	**LJ2**	**LJ3**	**LK1**	**LK2**
LF1	0	112	1	0	0	0	0	0	0	0
LF2		0	22	0	0	0	0	0	0	0
LF3			0	0	2	0	0	2	0	1
LI1				0	826	0	0	0	0	0
LI2					0	0	0	0	0	0
LJ1						0	31	0	0	0
LJ2							0	1	0	4
LJ3								0	0	27
LK1									0	0
LK2										0

*(A) Right side electrodes of interest. (B) Left side electrodes of interest*.

The electrodes with strong connections were all located in the perisylvian/opercular areas and the electrodes with weaker connections were in the middle and posterior short gyri of the anterior insula. This may be due to a number of reasons, for example: (1) the subject may have had reduced white matter connectivity from the insula due to the presence of epilepsy, or (2) the tracting program may not have been able to tract well from the insula due to the shape of the local anatomy. The medial edge of the insula is close to the thin gray matter sheet of the claustrum which contains its own connections (Crick and Koch, 2005). Tracts from the insula need to pass around it to reach many of the other brain structures.

General tracting in the patient brain was compared to the non-epileptic healthy brain. MRI data for 3 subjects were obtained from the Human Connectome Database (see http://www.humanconnectomeproject.org), matched for age (40–44 years old, 2 male). Diffusion data were collected with 64 directions at a spatial resolution of 1.5 mm^3^ and T1 images were acquired at 1 mm^3^ with a 3T Siemens MRI scanner. Tracting was performed between the insula gyri. The gyri were segmented by hand from the T1 images producing 5 regions of interest (ROIs) in each hemisphere, these were the short anterior gyrus (SAG), short middle gyrus (SMG), short posterior gyrus (SPG), long anterior gyrus (LAG), and long posterior gyrus (LPG). Diffusion data were processed as reported in section Imaging Data Acquisition and Processing above and tracting was performed from each gryrus to every other gyrus. The tract counts are summarized and compared in Table 5. Similar to the patient electrode study, few tracks were found between the right and left hemispheres (0–4 tracks) in controls and these results have been omitted for brevity.

**Table 5:** DTI tract counts between insula gyri for the patient, the mean of 3 controls and the calculated difference in the number of tracts.

**Gyri connection**	**Patient**	**Control mean**	**Difference relative to controls**
LSAG – LSMG	2,528	382	2,146
LSAG – LSPG	97	31	66
LSAG – LLAG	0	19	−19
LSAG – LLPG	2	34	−32
LSMG – LSPG	3,122	776	2,346
LSMG – LLAG	47	119	−72
LSMG – LLPG	0	5	−5
LSPG – LLAG	2,392	800	1,592
LSPG – LLPG	0	128	−128
LLAG – LLPG	22	391	−369
RSAG – RSMG	541	543	−2
RSAG – RSPG	3,648	74	3,574
RSAG – RLAG	156	98	58
RSAG – RLPG	0	74	−74
RSMG – RSPG	2,554	632	2,192
RSMG – RLAG	335	328	7
RSMG – RLPG	1	96	−95
RSPG – RLAG	3,359	939	2,420
RSPG – RLPG	137	51	86
RLAG – RLPG	714	313	401

Even with the difference in spatial resolution and number of diffusion directions between the control subject and the patient DTI data areas of greatly increased connectivity were found in the patient data compared to the mean values from the control data. These areas were: left side—short anterior ↔ short middle gyri, short middle ↔ short posterior gyri and short posterior ↔ long anterior gyri; right side—short anterior ↔ short posterior gyri, short middle ↔ short posterior gyri and short posterior ↔ long anterior gyri. The short posterior gyrus appears to have increased connectivity to the adjacent gyri (short middle and long anterior) in both hemispheres and may provide an underlying structural pathway for the propagation of the seizures. The bilateral aspect of the increased connectivity is particularly interesting in this patient with left insula epilepsy and suggests that the disease effects are not unilateral. Although not enough is known at this time about the implications of increased connectivity in seizure focus and propagation this information has the potential to aid clinicians in understanding the properties of epilepsy and inform the placement of the SEEG electrodes during presurgical planning.

## Discussion

The development of a workflow-based approach described in this paper to analyze the organization of brain functional connectivity networks during seizure events reveal that there is significant heterogeneity in terms of functional network motifs across seizures in a single patient. Further, integrative analysis of functional network with structural network information in epilepsy patients has significant advantages in terms of providing both spatial and temporal characteristics of the epileptic network. The directed graph motifs formed during seizure events show the formation of dominant network structures during the initial period of the seizure followed by their subsequent breakdown, which confirms findings of previous studies (Kramer et al., 2010). However, unlike previous studies the results reported in this study did not detect significant similarities between the graph motifs formed during a specific event across the multiple seizures recorded in this patient.

This may be due to multiple factors, including dosage of anti-epileptic drug (AED) (Vlooswijk et al., 2011), which may influence the functional networks formed during seizure events. The results from the hierarchical clustering represented as dendrograms showed interesting results across the 8 seizures. In particular, our data provides a new perspective regarding the distribution of similarity values between pair-wise electrode contacts and their neighboring electrode contacts across the baseline and seizure events. For example, our results show that during ictal 1 phase, the cosine similarity values between electrode pairs range from 1 (high similarity) to 0.16 (low similarity). This result shows that there is an overall increase in correlated interactions between brain regions during the seizure onset, which is statistically significant, followed by a trend toward decreased correlated interactions during subsequent ictal 1 and 2 phases. These findings are consistent with previously reported results, the strength of interactions is not consistently high but varies over a wide range of values. As far as we know, this detailed view of the similarity between electrode contacts during events have not been reported earlier.

In this work, the locations of the electrodes in and around the insula were used as ROIs to investigate white matter connections. It has the limitations of having few ROIs (< 20) and they cover only a few voxels in the DWI. In addition, they were also located in an area of the brain that is difficult to track from due to the proximity and shape of nearby structures. The result is a matrix that is too sparse to give meaningful cluster metrics in a similar way to the functional network measures. However, it is feasible to perform the tracking in other structures, or using larger ROIs or using a greater number of ROIs so that more informative measures about the structural network can be gained through a more stringent network analysis. Another factor that would improve the tractography is the acquisition of a higher quality diffusion weighted image with an increased number of directions and reduced voxel size compared to that used here.

Generally, these scanning factors equate to a longer scanning duration which would mean higher financial costs and time burden for both the patient and the radiology department but MRI technology is rapidly advancing. Hardware, such as multi-channel acquisition, can cut the scan time to one third making it possible for higher quality images to be acquired in less time.

The results generated from this integrative approach to analyze functional and structural network measures together during epileptic seizure events has many applications in epilepsy clinical care. In particular, large-scale comparative evaluation of structural and functional network measures in an epilepsy patient cohort can be used to support clinical decision making during pre-surgical evaluation of focal epilepsy patients. About 60% of epilepsy patients are diagnosed with focal epilepsy and many of these patients do not respond to anti-epileptic medication (Rosenow and Lüders, 2001). These pharmacoresistant patients are considered for surgery, which requires accurate delineation of the epileptogenic zone that protects the eloquent cortex. However, the delineation of the epileptogenic network is extremely challenging and poor delineation adversely affects the success of epilepsy surgery with 20–50% of patients experiencing recurrence of seizure after surgery (Englot et al., 2013). Development of effective methods for more accurate characterization of epileptogenic zone and epileptic seizure network is a significant research objective, which can be supported by using a workflow-based approach that also supports reproducibility. In addition, automation of multiple data processing and analysis steps will help address the challenge of processing large volumes of patient data. For example, many intermediate steps used to compute functional connectivity measures described in this paper are highly efficient and scalable (Sahoo et al., 2016).

A limitation of this study is that data is presented as a case study of one patient. Often clinically, patients must be evaluated on an individual basis and we wanted to show the utility of the workflow for a single subject. Additionally, data for a group analysis would need to be selectively chosen based on electrode location, seizure focus, and medical history to draw conclusions about structure and function in different types of epilepsy. An epileptic focus in the insula is relatively rare and further comparable datasets were not available at the time of this analysis.

## Summary

In this paper, we described the development and application of an integrative analysis technique to study epileptic seizure networks using functional as well as structural connectivity measures. The results from analysis of functional network connectivity during 27 events across 8 seizures show dissimilarities between the network structures formed during seizure events in a single patient. In particular, we used hierarchical clustering approach based on cosine similarity to characterize the formation of connected communities during various seizure events. Although the structural connectivity measures computed from DWI data were sparse due to lower resolution of imaging data generated during routine clinical care for this specific epilepsy patient, the integrative analysis approach developed in this paper has multiple applications in characterizing epileptic seizure networks. In addition, the use of a workflow-based approach for integrative analysis will allow analysis of seizure networks for large patient cohorts, which is likely to have appropriate statistical significance to support inference of association between structural and functional networks during seizure events.

## Data Availability Statement

The datasets generated for this study are available on request to the corresponding author.

## Ethics Statement

The studies involving human participants were reviewed and approved by University Hospitals Cleveland Medical Center (Approval Number: CR00001449). Written informed consent for participation was not required for this study in accordance with the national legislation and the institutional requirements.

## Author Contributions

SC and SS: study design, analysis and interpretation of data, and preparation of manuscript. AG: study design, analysis and interpretation of data, and review of manuscript. NS: collection, analysis, and interpretation of data and review of manuscript. SL: analysis and interpretation of data and review of manuscript. CT: study design, interpretation of data, and review of manuscript. All authors contributed to the article and approved the submitted version.

## Conflict of Interest

The authors declare that the research was conducted in the absence of any commercial or financial relationships that could be construed as a potential conflict of interest.
