# Supplementary material for: An Integrative Approach to Study Structural and Functional Network Connectivity in Epilepsy Using Imaging and Signal Data
Source: Front Integr Neurosci. 2021 Jan 12;14:491403. doi: 10.3389/fnint.2020.491403 (PMC7835283; doi:10.3389/fnint.2020.491403)
Supplement: Supplementary file 1 [file Data_Sheet_1.pdf]

Supplementary Data

To adjust for multiple comparisons, we employed the Benjamini-Hochberg (B-H) false discovery rate method. The original p-values and corresponding adjusted B-H p-value cutoffs are listed in the supplementary table. Results are interpreted relative to the adjusted thresholds (Tables 1-4).

Table 1:

			Seizure Onset		
Electrode Pair	P-Value (Sorted)	Rank	Averaged Rank	CriticalValue = (rank of p-value/105)*0.05	P-value < CV
RF1, LJ1	0.0078125	1	20	0.00952381	TRUE
RF2, LJ1	0.0078125	2	20	0.00952381	TRUE
RF3, LJ1	0.0078125	3	20	0.00952381	TRUE
RJ1, LJ1	0.0078125	4	20	0.00952381	TRUE
RJ2, LJ1	0.0078125	5	20	0.00952381	TRUE
LK1, LJ2	0.0078125	6	20	0.00952381	TRUE
RJ2, LJ2	0.0078125	7	20	0.00952381	TRUE
LF1, LJ3	0.0078125	8	20	0.00952381	TRUE
RF1, LJ3	0.0078125	9	20	0.00952381	TRUE
RF2, LJ3	0.0078125	10	20	0.00952381	TRUE
RF3, LJ3	0.0078125	11	20	0.00952381	TRUE
LK2, LK1	0.0078125	12	20	0.00952381	TRUE
LF1, LK1	0.0078125	13	20	0.00952381	TRUE
LF2, LK1	0.0078125	14	20	0.00952381	TRUE
LF3, LK1	0.0078125	15	20	0.00952381	TRUE
RJ1, LF1	0.0078125	16	20	0.00952381	TRUE
RF1, LF2	0.0078125	17	20	0.00952381	TRUE
RF2, LF2	0.0078125	18	20	0.00952381	TRUE
RF3, LF2	0.0078125	19	20	0.00952381	TRUE
RJ1, LF2	0.0078125	20	20	0.00952381	TRUE
RF1, LF3	0.0078125	21	20	0.00952381	TRUE
RF2, LF3	0.0078125	22	20	0.00952381	TRUE
RF3, LF3	0.0078125	23	20	0.00952381	TRUE
RJ1, LF3	0.0078125	24	20	0.00952381	TRUE
RJ1, RF1	0.0078125	25	20	0.00952381	TRUE
RJ2, RF1	0.0078125	26	20	0.00952381	TRUE
LI1, RF1	0.0078125	27	20	0.00952381	TRUE
LI2, RF1	0.0078125	28	20	0.00952381	TRUE
RJ1, RF2	0.0078125	29	20	0.00952381	TRUE
LI1, RF2	0.0078125	30	20	0.00952381	TRUE
LI2, RF2	0.0078125	31	20	0.00952381	TRUE
RJ1, RF3	0.0078125	32	20	0.00952381	TRUE
LI1, RF3	0.0078125	33	20	0.00952381	TRUE
LI2, RF3	0.0078125	34	20	0.00952381	TRUE
LI1, RJ1	0.0078125	35	20	0.00952381	TRUE
LI2, RJ1	0.0078125	36	20	0.00952381	TRUE
LI1, RJ2	0.0078125	37	20	0.00952381	TRUE
LI2, RJ2	0.0078125	38	20	0.00952381	TRUE
LI2, LI1	0.0078125	39	20	0.00952381	TRUE
LF1, LJ1	0.01171875	40	41.5	0.019761905	TRUE
RF1, LJ2	0.01171875	41	41.5	0.019761905	TRUE
RJ2, RF2	0.01171875	42	41.5	0.019761905	TRUE
RJ2, RF3	0.01171875	43	41.5	0.019761905	TRUE
LF3, LJ1	0.015625	44	46	0.021904762	TRUE
RF2, LJ2	0.015625	45	46	0.021904762	TRUE
LK1, LJ3	0.015625	46	46	0.021904762	TRUE
RJ2, LJ3	0.015625	47	46	0.021904762	TRUE
LF2, LF1	0.015625	48	46	0.021904762	TRUE
LJ3, LJ1	0.01953125	49	49	0.023333333	TRUE
RJ1, LJ2	0.0234375	50	50.5	0.024047619	TRUE
LI1, LF2	0.0234375	51	50.5	0.024047619	TRUE
RF1, LK2	0.02734375	52	52.5	0.025	FALSE
RF2, LF1	0.02734375	53	52.5	0.025	FALSE
LI2, LF2	0.03515625	54	54	0.025714286	FALSE
LJ3, LJ2	0.0390625	55	56	0.026666667	FALSE
LI1, LK1	0.0390625	56	56	0.026666667	FALSE
RF1, LF1	0.0390625	57	56	0.026666667	FALSE
LI2, LF3	0.046875	58	58	0.027619048	FALSE
LI2, LJ1	0.05078125	59	59	0.028095238	FALSE
LF2, LJ1	0.05859375	60	60.5	0.028809524	FALSE
LI2, LK1	0.05859375	61	60.5	0.028809524	FALSE
LK2, LJ2	0.078125	62	64	0.03047619	FALSE
LF1, LJ2	0.078125	63	64	0.03047619	FALSE
RF2, LK1	0.078125	64	64	0.03047619	FALSE
LI1, LF3	0.078125	65	64	0.03047619	FALSE
RF3, RF2	0.078125	66	64	0.03047619	FALSE
RJ2, LF2	0.08984375	67	67	0.031904762	FALSE
RF2, LK2	0.1328125	68	68	0.032380952	FALSE
LF1, LK2	0.13671875	69	69	0.032857143	FALSE
LI1, LJ1	0.15625	70	70	0.033333333	FALSE
LF3, LF1	0.1640625	71	71	0.033809524	FALSE
RF3, LK1	0.171875	72	72	0.034285714	FALSE
RF3, LJ2	0.17578125	73	73	0.034761905	FALSE
RJ1, LJ3	0.1796875	74	74	0.035238095	FALSE
LF2, LJ3	0.1875	75	75.5	0.035952381	FALSE
RF3, LF1	0.1875	76	75.5	0.035952381	FALSE
RF1, LK1	0.19140625	77	77	0.036666667	FALSE
LI1, LK2	0.21484375	78	78.5	0.037380952	FALSE
RJ2, LF3	0.21484375	79	78.5	0.037380952	FALSE
RJ2, LK2	0.22265625	80	80	0.038095238	FALSE
LK1, LJ1	0.25	81	81	0.038571429	FALSE
LF3, LJ2	0.28515625	82	82	0.039047619	FALSE
LI1, LF1	0.296875	83	83	0.03952381	FALSE
LF2, LJ2	0.3125	84	84	0.04	FALSE
RJ1, LK1	0.3203125	85	85	0.04047619	FALSE
RJ2, LK1	0.34765625	86	86.5	0.041190476	FALSE
LI2, LF1	0.34765625	87	86.5	0.041190476	FALSE
LK2, LJ3	0.375	88	88	0.041904762	FALSE
LK2, LJ1	0.41015625	89	89	0.042380952	FALSE
LF3, LJ3	0.41796875	90	90	0.042857143	FALSE
LF3, LF2	0.43359375	91	91	0.043333333	FALSE
LI2, LJ3	0.4375	92	92	0.043809524	FALSE
RJ2, LF1	0.4765625	93	93	0.044285714	FALSE
RJ2, RJ1	0.51171875	94	94	0.044761905	FALSE
RJ1, LK2	0.55859375	95	95	0.045238095	FALSE
LJ2, LJ1	0.5859375	96	96	0.045714286	FALSE
LF3, LK2	0.62890625	97	97	0.046190476	FALSE
LI2, LK2	0.6328125	98	98	0.046666667	FALSE
LI1, LJ3	0.65234375	99	99	0.047142857	FALSE
RF3, LK2	0.68359375	100	100	0.047619048	FALSE
LF2, LK2	0.7265625	101	101	0.048095238	FALSE
LI1, LJ2	0.734375	102	102	0.048571429	FALSE
LI2, LJ2	0.78515625	103	103	0.049047619	FALSE
RF2, RF1	0.83203125	104	104	0.04952381	FALSE
RF3, RF1	0.8828125	105	105	0.05	FALSE

Table 2:

			Ictal 1 Phase		
Electrode Pair	P-Value (Sorted)	Rank	Averaged Rank	CriticalValue = (rank of p-value/105)*0.05	P-value < CV
RJ1, RF3	0.0234375	1	1	0.00047619	FALSE
RJ1, RF2	0.03125	2	2	0.000952381	FALSE
RJ2, RF1	0.0390625	3	4.5	0.002142857	FALSE
LI1, RJ1	0.0390625	4	4.5	0.002142857	FALSE
LI2, RJ1	0.0390625	5	4.5	0.002142857	FALSE
LI1, RJ2	0.0390625	6	4.5	0.002142857	FALSE
LI2, RJ2	0.046875	7	7	0.003333333	FALSE
RJ1, RF1	0.0546875	8	8	0.003809524	FALSE
LI2, LI1	0.0703125	9	9	0.004285714	FALSE
RF1, LJ1	0.078125	10	11	0.005238095	FALSE
LI2, LF3	0.078125	11	11	0.005238095	FALSE
RJ2, RF2	0.078125	12	11	0.005238095	FALSE
LI2, RF1	0.08984375	13	13	0.006190476	FALSE
LJ2, LJ1	0.1015625	14	14.5	0.006904762	FALSE
RJ2, LJ1	0.1015625	15	14.5	0.006904762	FALSE
RJ1, LJ1	0.109375	16	16.5	0.007857143	FALSE
RJ2, RF3	0.109375	17	16.5	0.007857143	FALSE
LI1, RF1	0.12109375	18	18	0.008571429	FALSE
RF2, LJ1	0.1640625	19	19.5	0.009285714	FALSE
RF3, LF2	0.1640625	20	19.5	0.009285714	FALSE
LI2, LF2	0.17578125	21	21	0.01	FALSE
RJ1, LF3	0.18359375	22	22	0.01047619	FALSE
RJ1, LF2	0.2109375	23	23	0.010952381	FALSE
RF2, LF3	0.21484375	24	24	0.011428571	FALSE
RF3, LF3	0.21875	25	25	0.011904762	FALSE
RF2, LF2	0.25	26	26	0.012380952	FALSE
LI1, LF3	0.265625	27	27	0.012857143	FALSE
RJ2, LK1	0.296875	28	29.5	0.014047619	FALSE
LF3, LF2	0.296875	29	29.5	0.014047619	FALSE
RF3, RF2	0.296875	30	29.5	0.014047619	FALSE
LI2, RF2	0.296875	31	29.5	0.014047619	FALSE
LK2, LK1	0.3125	32	32	0.015238095	FALSE
RF1, LJ3	0.328125	33	33.5	0.015952381	FALSE
RF1, LK1	0.328125	34	33.5	0.015952381	FALSE
LK2, LJ3	0.33203125	35	36.5	0.017380952	FALSE
RF2, LK1	0.33203125	36	36.5	0.017380952	FALSE
LI1, LF2	0.33203125	37	36.5	0.017380952	FALSE
LI1, RF3	0.33203125	38	36.5	0.017380952	FALSE
RF3, LJ1	0.33984375	39	39	0.018571429	FALSE
LK1, LJ3	0.359375	40	40.5	0.019285714	FALSE
LI2, LK1	0.359375	41	40.5	0.019285714	FALSE
LI1, LK1	0.36328125	42	42	0.02	FALSE
RF2, LJ3	0.3671875	43	44.5	0.021190476	FALSE
RJ1, LK1	0.3671875	44	44.5	0.021190476	FALSE
LI1, RF2	0.3671875	45	44.5	0.021190476	FALSE
LI2, RF3	0.3671875	46	44.5	0.021190476	FALSE
RF1, LJ2	0.3984375	47	47	0.022380952	FALSE
LK2, LJ1	0.40234375	48	48	0.022857143	FALSE
RF3, LK1	0.4375	49	49	0.023333333	FALSE
LI2, LJ1	0.44140625	50	50	0.023809524	FALSE
LF1, LJ2	0.4453125	51	51	0.024285714	FALSE
LF3, LJ1	0.4765625	52	52	0.024761905	FALSE
LJ3, LJ1	0.51953125	53	53	0.025238095	FALSE
LI1, LJ1	0.5234375	54	54	0.025714286	FALSE
LF3, LJ3	0.53125	55	55	0.026190476	FALSE
LF1, LJ1	0.56640625	56	56.5	0.026904762	FALSE
RF3, LJ3	0.56640625	57	56.5	0.026904762	FALSE
RF1, LF3	0.5703125	58	58	0.027619048	FALSE
LF2, LJ1	0.57421875	59	60	0.028571429	FALSE
LF1, LK2	0.57421875	60	60	0.028571429	FALSE
RF3, LF1	0.57421875	61	60	0.028571429	FALSE
LF3, LJ2	0.578125	62	62	0.02952381	FALSE
LI1, LK2	0.6171875	63	63.5	0.030238095	FALSE
RF1, LF2	0.6171875	64	63.5	0.030238095	FALSE
LI2, LJ2	0.625	65	65	0.030952381	FALSE
RJ2, LF3	0.62890625	66	66	0.031428571	FALSE
LK1, LJ2	0.66015625	67	67.5	0.032142857	FALSE
LK2, LJ2	0.66015625	68	67.5	0.032142857	FALSE
LF2, LJ2	0.7109375	69	70.5	0.033571429	FALSE
RF3, LJ2	0.7109375	70	70.5	0.033571429	FALSE
RJ2, LJ2	0.7109375	71	70.5	0.033571429	FALSE
RJ1, LK2	0.7109375	72	70.5	0.033571429	FALSE
RJ1, LJ2	0.71875	73	74	0.035238095	FALSE
LF1, LJ3	0.71875	74	74	0.035238095	FALSE
LF1, LK1	0.71875	75	74	0.035238095	FALSE
LF3, LK2	0.72265625	76	76	0.036190476	FALSE
LI2, LJ3	0.7265625	77	78	0.037142857	FALSE
RF3, LK2	0.7265625	78	78	0.037142857	FALSE
RF3, RF1	0.7265625	79	78	0.037142857	FALSE
LI1, LJ2	0.76953125	80	80.5	0.038333333	FALSE
RJ2, LF1	0.76953125	81	80.5	0.038333333	FALSE
LI1, LJ3	0.8125	82	83	0.03952381	FALSE
RF2, LK2	0.8125	83	83	0.03952381	FALSE
RF1, LF1	0.8125	84	83	0.03952381	FALSE
LI1, LF1	0.81640625	85	85.5	0.040714286	FALSE
RF2, RF1	0.81640625	86	85.5	0.040714286	FALSE
LF2, LJ3	0.8203125	87	87.5	0.041666667	FALSE
LF2, LF1	0.8203125	88	87.5	0.041666667	FALSE
LJ3, LJ2	0.85546875	89	90.5	0.043095238	FALSE
RF1, LK2	0.85546875	90	90.5	0.043095238	FALSE
RF2, LF1	0.85546875	91	90.5	0.043095238	FALSE
RJ1, LF1	0.85546875	92	90.5	0.043095238	FALSE
LF3, LK1	0.86328125	93	93.5	0.04452381	FALSE
RJ2, LF2	0.86328125	94	93.5	0.04452381	FALSE
RJ1, LJ3	0.90625	95	96	0.045714286	FALSE
LF3, LF1	0.90625	96	96	0.045714286	FALSE
LI2, LF1	0.90625	97	96	0.045714286	FALSE
RJ2, LJ3	0.91015625	98	98	0.046666667	FALSE
LK1, LJ1	0.9140625	99	100	0.047619048	FALSE
RJ2, LK2	0.9140625	100	100	0.047619048	FALSE
LI2, LK2	0.9140625	101	100	0.047619048	FALSE
RF2, LJ2	0.9609375	102	102.5	0.048809524	FALSE
RJ2, RJ1	0.9609375	103	102.5	0.048809524	FALSE
LF2, LK1	1	104	104.5	0.049761905	FALSE
LF2, LK2	1	105	104.5	0.049761905	FALSE

Table 3:

			Ictal 2 Phase		
Electrode Pair	P-Value (Sorted)	Rank	Averaged Rank	CriticalValue = (rank of p-value/105)*0.05	P-value < CV
LF1, LJ1	0.015625	1	15.5	0.007380952	FALSE
RF1, LJ1	0.015625	2	15.5	0.007380952	FALSE
RF2, LJ1	0.015625	3	15.5	0.007380952	FALSE
RF3, LJ1	0.015625	4	15.5	0.007380952	FALSE
RJ1, LJ1	0.015625	5	15.5	0.007380952	FALSE
RJ2, LJ1	0.015625	6	15.5	0.007380952	FALSE
LI1, LJ1	0.015625	7	15.5	0.007380952	FALSE
LI2, LJ1	0.015625	8	15.5	0.007380952	FALSE
LK2, LK1	0.015625	9	15.5	0.007380952	FALSE
LF1, LK1	0.015625	10	15.5	0.007380952	FALSE
RF1, LK1	0.015625	11	15.5	0.007380952	FALSE
RF2, LK1	0.015625	12	15.5	0.007380952	FALSE
RF3, LK1	0.015625	13	15.5	0.007380952	FALSE
RJ1, LK1	0.015625	14	15.5	0.007380952	FALSE
LI1, LK1	0.015625	15	15.5	0.007380952	FALSE
LI2, LK1	0.015625	16	15.5	0.007380952	FALSE
LF1, LK2	0.015625	17	15.5	0.007380952	FALSE
RJ1, LF2	0.015625	18	15.5	0.007380952	FALSE
RJ1, LF3	0.015625	19	15.5	0.007380952	FALSE
RJ1, RF1	0.015625	20	15.5	0.007380952	FALSE
RJ2, RF1	0.015625	21	15.5	0.007380952	FALSE
LI2, RF1	0.015625	22	15.5	0.007380952	FALSE
RJ1, RF2	0.015625	23	15.5	0.007380952	FALSE
LI2, RF2	0.015625	24	15.5	0.007380952	FALSE
RJ1, RF3	0.015625	25	15.5	0.007380952	FALSE
LI2, RF3	0.015625	26	15.5	0.007380952	FALSE
LI1, RJ1	0.015625	27	15.5	0.007380952	FALSE
LI2, RJ1	0.015625	28	15.5	0.007380952	FALSE
LI1, RJ2	0.015625	29	15.5	0.007380952	FALSE
LI2, RJ2	0.015625	30	15.5	0.007380952	FALSE
LI2, LK2	0.0234375	31	32	0.015238095	FALSE
RJ2, RF2	0.0234375	32	32	0.015238095	FALSE
RJ2, RF3	0.0234375	33	32	0.015238095	FALSE
LF2, LJ1	0.03125	34	39.5	0.018809524	FALSE
LK1, LJ2	0.03125	35	39.5	0.018809524	FALSE
LK1, LJ3	0.03125	36	39.5	0.018809524	FALSE
LI1, LK2	0.03125	37	39.5	0.018809524	FALSE
RF2, LF2	0.03125	38	39.5	0.018809524	FALSE
LI2, LF2	0.03125	39	39.5	0.018809524	FALSE
RF1, LF3	0.03125	40	39.5	0.018809524	FALSE
RF2, LF3	0.03125	41	39.5	0.018809524	FALSE
RF3, LF3	0.03125	42	39.5	0.018809524	FALSE
LI1, RF1	0.03125	43	39.5	0.018809524	FALSE
LI1, RF2	0.03125	44	39.5	0.018809524	FALSE
LI1, RF3	0.03125	45	39.5	0.018809524	FALSE
LK1, LJ1	0.0390625	46	47	0.022380952	FALSE
RF3, LF2	0.0390625	47	47	0.022380952	FALSE
LI2, LF3	0.0390625	48	47	0.022380952	FALSE
LF3, LJ1	0.046875	49	50	0.023809524	FALSE
LF3, LK1	0.046875	50	50	0.023809524	FALSE
LF3, LF2	0.046875	51	50	0.023809524	FALSE
RJ2, LK1	0.0546875	52	52.5	0.025	FALSE
RF2, LK2	0.0546875	53	52.5	0.025	FALSE
LF3, LJ2	0.0625	54	54.5	0.025952381	FALSE
LF2, LK1	0.0625	55	54.5	0.025952381	FALSE
RF3, LK2	0.0703125	56	56	0.026666667	FALSE
LJ3, LJ1	0.078125	57	58.5	0.027857143	FALSE
LF2, LJ2	0.078125	58	58.5	0.027857143	FALSE
RF1, LF2	0.078125	59	58.5	0.027857143	FALSE
RJ2, LF3	0.078125	60	58.5	0.027857143	FALSE
LF3, LK2	0.0859375	61	61	0.029047619	FALSE
LF2, LJ3	0.1015625	62	62	0.02952381	FALSE
LF2, LK2	0.109375	63	63.5	0.030238095	FALSE
LI1, LF2	0.109375	64	63.5	0.030238095	FALSE
LF1, LJ2	0.1171875	65	66	0.031428571	FALSE
LI2, LJ2	0.1171875	66	66	0.031428571	FALSE
RJ1, LF1	0.1171875	67	66	0.031428571	FALSE
RJ2, LF2	0.1328125	68	68	0.032380952	FALSE
RF3, LJ3	0.140625	69	70	0.033333333	FALSE
RF1, LK2	0.140625	70	70	0.033333333	FALSE
LI1, LF3	0.140625	71	70	0.033333333	FALSE
RF2, LJ2	0.15625	72	72.5	0.03452381	FALSE
RF2, LJ3	0.15625	73	72.5	0.03452381	FALSE
LI1, LJ2	0.1796875	74	74.5	0.03547619	FALSE
LI2, LJ3	0.1796875	75	74.5	0.03547619	FALSE
RF3, RF2	0.203125	76	76	0.036190476	FALSE
RF3, LJ2	0.2265625	77	77.5	0.036904762	FALSE
RF1, LJ3	0.2265625	78	77.5	0.036904762	FALSE
LF3, LF1	0.2421875	79	79	0.037619048	FALSE
LI1, LJ3	0.2578125	80	80	0.038095238	FALSE
LI2, LF1	0.2734375	81	81	0.038571429	FALSE
RF1, LJ2	0.328125	82	82	0.039047619	FALSE
LI1, LF1	0.34375	83	83.5	0.039761905	FALSE
RJ2, RJ1	0.34375	84	83.5	0.039761905	FALSE
LI2, LI1	0.359375	85	85	0.04047619	FALSE
LF3, LJ3	0.3984375	86	86	0.040952381	FALSE
RF3, LF1	0.4140625	87	87	0.041428571	FALSE
LF2, LF1	0.4375	88	88	0.041904762	FALSE
LK2, LJ2	0.453125	89	89	0.042380952	FALSE
RJ2, LJ2	0.4921875	90	90	0.042857143	FALSE
LF1, LJ3	0.5546875	91	91	0.043333333	FALSE
LJ2, LJ1	0.640625	92	92	0.043809524	FALSE
LK2, LJ1	0.65625	93	94	0.044761905	FALSE
LK2, LJ3	0.65625	94	94	0.044761905	FALSE
RJ2, LJ3	0.65625	95	94	0.044761905	FALSE
RJ2, LF1	0.6640625	96	96	0.045714286	FALSE
RJ1, LJ3	0.7109375	97	97	0.046190476	FALSE
RF3, RF1	0.71875	98	98	0.046666667	FALSE
RF1, LF1	0.7265625	99	99	0.047142857	FALSE
RF2, LF1	0.7734375	100	100	0.047619048	FALSE
RJ1, LJ2	0.78125	101	101	0.048095238	FALSE
RF2, RF1	0.84375	102	102	0.048571429	FALSE
LJ3, LJ2	0.90625	103	103	0.049047619	FALSE
RJ1, LK2	0.921875	104	104	0.04952381	FALSE
RJ2, LK2	0.9609375	105	105	0.05	FALSE

Table 4:

			Ictal 3 Phase		
Electrode Pair	P-Value (Sorted)	Rank	Averaged Rank	CriticalValue = (rank of p-value/105)*0.05	P-value < CV
LJ2, LJ1	0.125	1	35.5	0.016904762	FALSE
LJ3, LJ1	0.125	2	35.5	0.016904762	FALSE
RF1, LJ1	0.125	3	35.5	0.016904762	FALSE
RF2, LJ1	0.125	4	35.5	0.016904762	FALSE
RF3, LJ1	0.125	5	35.5	0.016904762	FALSE
RJ1, LJ1	0.125	6	35.5	0.016904762	FALSE
RJ2, LJ1	0.125	7	35.5	0.016904762	FALSE
LJ3, LJ2	0.125	8	35.5	0.016904762	FALSE
LK2, LJ2	0.125	9	35.5	0.016904762	FALSE
RF3, LJ2	0.125	10	35.5	0.016904762	FALSE
RJ2, LJ2	0.125	11	35.5	0.016904762	FALSE
LI1, LJ2	0.125	12	35.5	0.016904762	FALSE
LI2, LJ2	0.125	13	35.5	0.016904762	FALSE
LK2, LJ3	0.125	14	35.5	0.016904762	FALSE
LF2, LJ3	0.125	15	35.5	0.016904762	FALSE
RF1, LJ3	0.125	16	35.5	0.016904762	FALSE
RF2, LJ3	0.125	17	35.5	0.016904762	FALSE
RF3, LJ3	0.125	18	35.5	0.016904762	FALSE
LI1, LJ3	0.125	19	35.5	0.016904762	FALSE
LI2, LJ3	0.125	20	35.5	0.016904762	FALSE
LK2, LK1	0.125	21	35.5	0.016904762	FALSE
LF2, LK1	0.125	22	35.5	0.016904762	FALSE
LF3, LK1	0.125	23	35.5	0.016904762	FALSE
RF1, LK1	0.125	24	35.5	0.016904762	FALSE
RF2, LK1	0.125	25	35.5	0.016904762	FALSE
RF3, LK1	0.125	26	35.5	0.016904762	FALSE
LI1, LK1	0.125	27	35.5	0.016904762	FALSE
LI2, LK1	0.125	28	35.5	0.016904762	FALSE
RF3, LK2	0.125	29	35.5	0.016904762	FALSE
RJ1, LK2	0.125	30	35.5	0.016904762	FALSE
RJ2, LK2	0.125	31	35.5	0.016904762	FALSE
LI1, LK2	0.125	32	35.5	0.016904762	FALSE
LI2, LK2	0.125	33	35.5	0.016904762	FALSE
LF2, LF1	0.125	34	35.5	0.016904762	FALSE
LF3, LF1	0.125	35	35.5	0.016904762	FALSE
LI1, LF1	0.125	36	35.5	0.016904762	FALSE
LI2, LF1	0.125	37	35.5	0.016904762	FALSE
LF3, LF2	0.125	38	35.5	0.016904762	FALSE
RF1, LF2	0.125	39	35.5	0.016904762	FALSE
RF2, LF2	0.125	40	35.5	0.016904762	FALSE
RF3, LF2	0.125	41	35.5	0.016904762	FALSE
RJ1, LF2	0.125	42	35.5	0.016904762	FALSE
RJ2, LF2	0.125	43	35.5	0.016904762	FALSE
LI1, LF2	0.125	44	35.5	0.016904762	FALSE
LI2, LF2	0.125	45	35.5	0.016904762	FALSE
RF1, LF3	0.125	46	35.5	0.016904762	FALSE
RF2, LF3	0.125	47	35.5	0.016904762	FALSE
RF3, LF3	0.125	48	35.5	0.016904762	FALSE
RJ1, LF3	0.125	49	35.5	0.016904762	FALSE
RJ2, LF3	0.125	50	35.5	0.016904762	FALSE
LI1, LF3	0.125	51	35.5	0.016904762	FALSE
LI2, LF3	0.125	52	35.5	0.016904762	FALSE
RJ1, RF1	0.125	53	35.5	0.016904762	FALSE
RJ2, RF1	0.125	54	35.5	0.016904762	FALSE
LI1, RF1	0.125	55	35.5	0.016904762	FALSE
LI2, RF1	0.125	56	35.5	0.016904762	FALSE
RF3, RF2	0.125	57	35.5	0.016904762	FALSE
RJ1, RF2	0.125	58	35.5	0.016904762	FALSE
RJ2, RF2	0.125	59	35.5	0.016904762	FALSE
LI1, RF2	0.125	60	35.5	0.016904762	FALSE
LI2, RF2	0.125	61	35.5	0.016904762	FALSE
RJ1, RF3	0.125	62	35.5	0.016904762	FALSE
RJ2, RF3	0.125	63	35.5	0.016904762	FALSE
LI1, RF3	0.125	64	35.5	0.016904762	FALSE
LI2, RF3	0.125	65	35.5	0.016904762	FALSE
RJ2, RJ1	0.125	66	35.5	0.016904762	FALSE
LI1, RJ1	0.125	67	35.5	0.016904762	FALSE
LI2, RJ1	0.125	68	35.5	0.016904762	FALSE
LI1, RJ2	0.125	69	35.5	0.016904762	FALSE
LI2, RJ2	0.125	70	35.5	0.016904762	FALSE
LF3, LJ3	0.1875	71	72	0.034285714	FALSE
RF2, LK2	0.1875	72	72	0.034285714	FALSE
LI2, LI1	0.1875	73	72	0.034285714	FALSE
LF2, LJ2	0.25	74	75.5	0.035952381	FALSE
RJ2, LJ3	0.25	75	75.5	0.035952381	FALSE
LF2, LK2	0.25	76	75.5	0.035952381	FALSE
RF3, RF1	0.25	77	75.5	0.035952381	FALSE
LF2, LJ1	0.3125	78	79.5	0.037857143	FALSE
LI2, LJ1	0.3125	79	79.5	0.037857143	FALSE
LF3, LJ2	0.3125	80	79.5	0.037857143	FALSE
RF2, LJ2	0.3125	81	79.5	0.037857143	FALSE
LK1, LJ1	0.375	82	83.5	0.039761905	FALSE
LI1, LJ1	0.375	83	83.5	0.039761905	FALSE
LF1, LJ2	0.375	84	83.5	0.039761905	FALSE
RJ2, LK1	0.375	85	83.5	0.039761905	FALSE
LK1, LJ3	0.5	86	88	0.041904762	FALSE
LF1, LK1	0.5	87	88	0.041904762	FALSE
LF1, LK2	0.5	88	88	0.041904762	FALSE
LF3, LK2	0.5	89	88	0.041904762	FALSE
RF3, LF1	0.5	90	88	0.041904762	FALSE
RF1, LK2	0.5625	91	91	0.043333333	FALSE
RJ1, LJ2	0.625	92	93.5	0.04452381	FALSE
LF1, LJ3	0.625	93	93.5	0.04452381	FALSE
RF1, LF1	0.625	94	93.5	0.04452381	FALSE
RJ2, LF1	0.625	95	93.5	0.04452381	FALSE
RJ1, LF1	0.6875	96	96	0.045714286	FALSE
LK2, LJ1	0.75	97	98	0.046666667	FALSE
LF3, LJ1	0.75	98	98	0.046666667	FALSE
RJ1, LK1	0.75	99	98	0.046666667	FALSE
RF1, LJ2	0.8125	100	100.5	0.047857143	FALSE
RF2, LF1	0.8125	101	100.5	0.047857143	FALSE
RJ1, LJ3	0.875	102	102.5	0.048809524	FALSE
RF2, RF1	0.875	103	102.5	0.048809524	FALSE
LF1, LJ1	0.9375	104	104	0.04952381	FALSE
LK1, LJ2	1	105	105	0.05	FALSE
