# Supplementary figures and images for: An Integrative Approach to Study Structural and Functional Network Connectivity in Epilepsy Using Imaging and Signal Data
Source: Front Integr Neurosci. 2021 Jan 12;14:491403. doi: 10.3389/fnint.2020.491403 (PMC7835283; doi:10.3389/fnint.2020.491403)

Count (Electrode Contact Pairs)

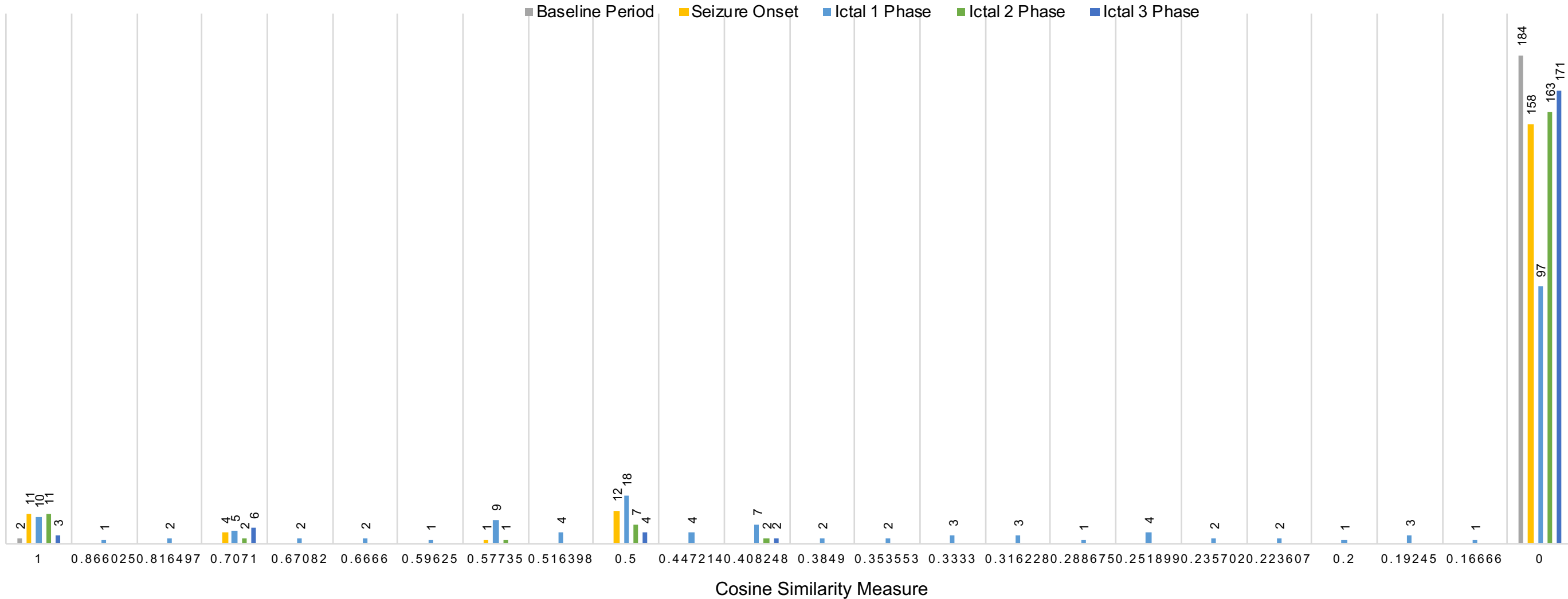

Supplement: Supplementary file 2 [file Data_Sheet_2.PDF]
